# A transient self-assembling self-replicator

**DOI:** 10.1038/s41467-018-04670-2

**Published:** 2018-06-08

**Authors:** Ignacio Colomer, Sarah M. Morrow, Stephen P. Fletcher

**Affiliations:** Department of Chemistry, Chemistry Research Laboratory, University of Oxford, 12 Mansfield Road, Oxford, OX1 3TA UK

## Abstract

Developing physical models of complex dynamic systems showing emergent behaviour is key to informing on persistence and replication in biology, how living matter emerges from chemistry, and how to design systems with new properties. Herein we report a fully synthetic small molecule system in which a surfactant replicator is formed from two phase-separated reactants using an alkene metathesis catalyst. The replicator self-assembles into aggregates, which catalyse their own formation, and is thermodynamically unstable. Rather than replicating until the reactants are fully consumed, the metastable replicator is depleted in a second metathesis reaction, and closed system equilibrium is eventually reached. Mechanistic experiments suggest phase separation is responsible for both replicator formation and destruction.

## Abstract (web summary)

Understanding self-replication and persistence in an out-of-equilibrium state is key to designing systems with new properties mimicking “living systems”. Here, the authors developed a synthetic small molecule system in which a transient surfactant replicator is responsible for both an autocatalytic aggregation pathway and a destructive pathway.

## Introduction

Understanding the basic principles and properties that govern living systems is a first step towards the creation of synthetic life. Many have concluded that a combination of standard physical laws and new concepts are required to understand what separates biological and simpler chemical matter^1–4^. While thermodynamic stability and the Second Law of Thermodynamics drive chemical processes, additional considerations are required for the richer, dynamic behaviour of life-like systems. However, understanding how energy is consumed, used, and dissipated to drive biological assembly and function remains challenging^5–7^. A synthetic model of a living system would likely be held out-of-equilibrium by the consumption of energy, and crucially involve self-replication to produce a persistent replicator population which could evolve^8^. Strategies to develop far-from-equilibrium processes resembling functions in living systems, such as dissipative self-assembly to mimic guanosine triphosphate-driven microtubule assembly, have also recently emerged^9,10^. Other dissipative self-assembling structures have also been reported^11–15^, including the generation of transient fibres whose formation/destruction occurs concurrently^16,17^.

Achieving non-biological self-replication alone in small molecule systems is non-trivial and has given rise to several approaches^18^. Artificial self-replication induced by templates has been observed in small molecules capable of recognition^19,20^ and the formation of large supramolecular aggregates^21,22^ in which the products act as seeds for their own formation. Systems based on physical replication, where aggregates of an amphiphilic product catalyse an interfacial reaction to form their own components^23,24^ have been explored from an origins-of-life perspective as this replication mechanism forms compartments which allow for localisation and enclosure of chemical components and reactions. In the cases described so far, the most efficient replicators come to dominate the system to yield either the thermodynamic or kinetically trapped products.

**Fig. 1:**
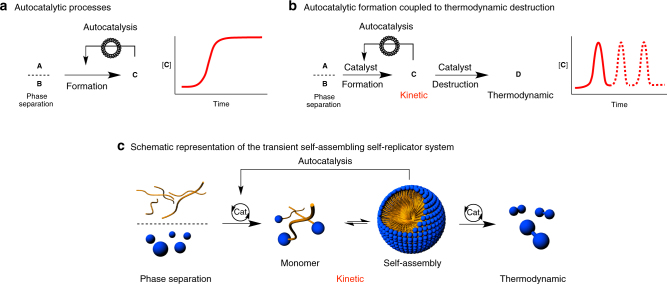
Examples of autocatalysis. **a** An autocatalytic system based on phase separation. **b** An autocatalytic system based on phase separation, coupled to thermodynamic destruction, that in a closed set-up experiment will evolve towards thermodynamic equilibrium. **c** Schematic representation of a transient self-assembling self-replicator system

Replicators that are simultaneously created and destroyed may help us to understand living systems^25,26^, which appear to work on similar principles. Simple physical autocatalytic systems are based on bond-formation or bond-cleavage between two phase-separated reactants **A** and **B**, one hydrophilic and one hydrophobic, that react across the interface to generate an amphiphilic product **C** (Fig. 1a). Product **C**, which self-assembles, typically shows a lag period followed by exponential growth, because the assembled structure accelerates the rate of the reaction, therefore acting as an autocatalyst^18,27^. Eventually the starting materials are consumed, there is a maximum in the concentration of product **C**, and while the system shows non-linear behaviour during its exponential phase it continuously moves towards and eventually reaches thermodynamic equilibrium and is essentially “dead”.

Here, we report a system that uses phase behaviour to drive the self-reproduction of minimal cell-like aggregates. The aggregates form by the self-assembly of non-thermodynamically stable amphiphiles produced using a metathesis catalyst. The catalyst also destroys the amphiphiles to form thermodynamic products. Understanding these processes may be relevant to biological systems, which appear to work on similar principles. Our system also consists of two reactants **A** and **B** that give amphiphilic **C**, which self-assembles. However, in this case **C** is also consumed to form a thermodynamically stable waste product **D**, so that the reaction profile shows formation of the product to a maximum concentration, followed by depletion of the product until all of **A** and **C** have been consumed (Fig. 1b, solid red line). The exponential phase of this reaction therefore moves away from the equilibrium state, in contrast to classical autocatalytic reactions. The energy and instability of the functional self-replicator **C** allows temporal control over the presence and function of the self-replicator. When starting materials or “food” (or “fuel”) are resupplied to the reaction mixture in a second or third batch (Fig. 1b, hashed red lines), the replicator may be reformed before again degrading to product **D**. Here the self-reproducing species **C** exists in a stage where it has some “life-like” characteristics and, like a variety of simple single cell organisms, the persistence and function of the replicator can be controlled by a supply of reactants (Fig. 1c).

## Results

### Characterisation of the system

**Fig. 2:**
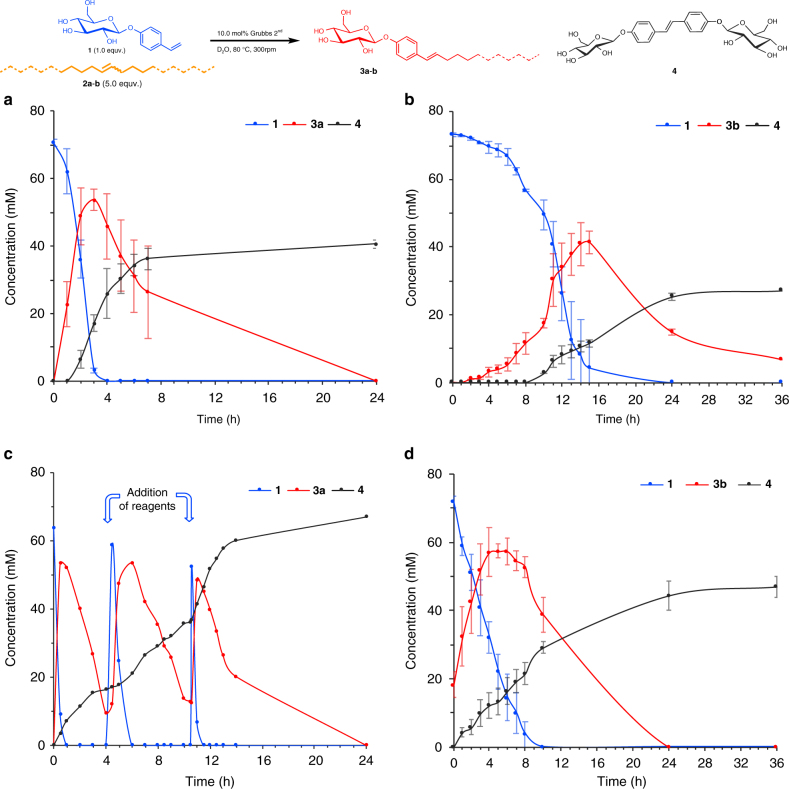
Kinetics of the self-replicating system showing concentration versus time of **1** (blue), **3** (red) and **4** (black). **a** The reaction of hydrophobic alkene **2a** with a shorter carbon chain, shows autocatalytic kinetics in the consumption of **1** and formation of **3a**. After reaching a maximum, thermodynamically unstable product **3a** is consumed to form waste product **4**. **b** The reaction of hydrophobic alkene **2b** with a longer carbon chain again shows autocatalytic kinetics with a longer lag-period on account of the reduced water miscibility of alkene **2b**. The peak in the concentration of **3b** is again followed by its consumption to form waste product **4**. **c** Re-fuelling starting materials **1** and **2a**, once **3a** is depleted, allows for the self-replication to resume. An increased stirring rate is used to increase the rate of the reaction between **1** and **2a**, but the same pattern observed in Fig. 2a emerges. **d** Reaction of hydrophobic alkene **2b**, seeding with 20 mol% of surfactant **3b**. In this case waste product **4** is formed from the beginning as the metastable replictator is already present. Error bars represent standard deviation obtained from three repetitions

Key to the operation of our system is a catalyst that is capable of both making and breaking new covalent bonds. We chose to use a ruthenium-catalysed alkene cross-metathesis approach for which, as far as we are aware, there is no known analogous reactivity in living systems. However, Ru-mediated metathesis is generally robust and versatile, and compatible with water^28–30^. Using hydrophilic alkene **1** and hydrophobic alkenes **2a–b**, which are phase separated (Fig. 2), and Grubbs 2nd generation catalyst in D_2_O we studied the evolution of the different species generated with hydrophobic alkenes **2a** (Fig. 2a) and **2b** (Fig. 2b), which differ only by their carbon chain lengths. Both alkenes show similar behaviour, displaying sigmoidal kinetics characteristic of autocatalytic systems^18,27^. The initial lag period is much longer for hydrophobic alkene **2b**, probably due to its reduced water miscibility (Fig. 2a vs. 2b). While consumption of hydrophilic alkene **1** shows classical sigmoidal autocatalytic behaviour (see blue line in Fig. 2b), the formation of amphiphilic **3** shows exponential growth with an initial lag period. After reaching a maximum point, **3** then gets completely consumed (see red line in Fig. 2b). The decomposition of replicator **3** gives soluble **4**, which we believe is the thermodynamic product. Interestingly, formation of **4** does not occur from the beginning of the reaction, but only when a significant concentration of amphiphile **3** is reached and hydrophilic alkene **1** is almost consumed (see Fig. 2a, b). This would imply that only when the surfactant is self-assembled is it destroyed, in analogy to the disassembly of microtubules, in which it is the association of tubulin-GTP units in the supramolecular structure which activates the hydrolysis and eventual disassembly of the entire structure^17,31^.

To demonstrate the potential for regeneration of the self-replicator, the reaction between **1** and **2a** was run in batch, and then once replicator **3a** had almost been fully consumed, starting materials **1** and **2a** were resupplied (Fig. 2c). These experiments show that a population of replicator **3a** can be regenerated before the inevitable conversion to product **4**, and this process was repeated a third time without fatigue. Moreover, these experiments show the potential to control the self replicator population and maintain a far-from-equilibrium state of the metastable surfactant self-replicator.

The autocatalytic behaviour of **3b** was further studied by performing seeding experiments, where addition of amphiphile **3b** from the beginning of the reaction suppressed the initial lag-period in both consumption of starting material **1** and formation of cross-product **3b** (Fig. 2d and Supplementary Fig. 14).

**Fig. 3:**
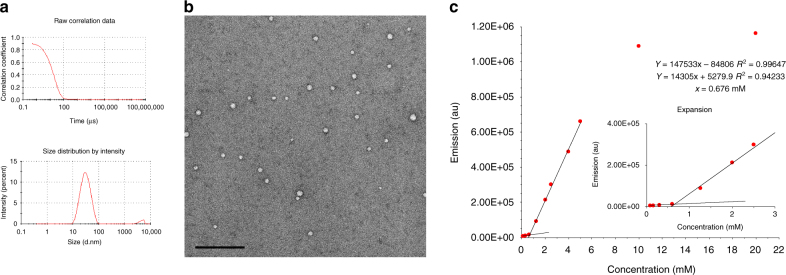
Characterisation of self-assembled amphiphile **3a**. **a** DLS shows good correlation with an average size particle of 28 nm. **b** TEM image showing particles with an average diameter of 27 nm (scale bar of 200 nm). **c** Emission vs. concentration and CMC determination

The aggregation properties of the amphiphiles **3a–b** were studied using dynamic light scattering (DLS) and transmission electron microscopy (TEM), and the critical micelle concentration (CMC) was determined using an established fluorimetric method (Fig. 3 and Supplementary Fig. 16–21).

### Mechanistic experiments

**Fig. 4:**
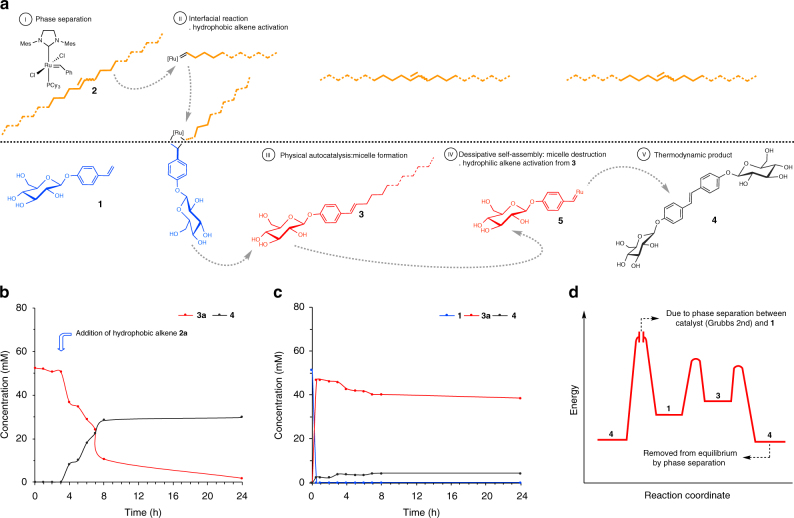
Mechanistic hypothesis supported by control experiments. **a** Diagram of the dynamic system including the chemical structures involved. Kinetic analysis representing concentration vs. time of **1** (blue), **3a** (red) and **4** (black) for the control experiments: **b** In D_2_O replicator **3a** is stable. It is only on addition of alkene **2a** that it is fully consumed to form waste product **4**. **c** In reaction between **1** and **2a** performed under homogeneous conditions no autocatalytic kinetics are observed for the formation of **3a**. Under these conditions product **3a** appears to be relatively stable. **d** Qualitative energy profile of the system, highlighting the higher effective barrier for direct conversion of **1** to **4** under phase separated conditions. Phase separation may shift the equilibrium position between **3a** and **4** by effectively removing water soluble **4** from the micellar environment

In order to probe the reaction pathways that operate in this complex biphasic system, which will inform on the design of subsequent far-from-equilibrium self-replicators, and help us to better understand what features of the current system are necessary for its successful operation, we carried out a series of control experiments (Fig. 4). What emerges from the experiments described below is that, at least for the current system described here, the biphasic nature of the reaction medium, and a flow of energy from starting materials to products are both key for allowing concurrent formation and destruction steps, and, therefore, the far-from-equilibrium state.

Mechanistically, we propose that reactions initiate in the organic phase (I—Phase separation in Fig. 4a), where the ruthenium catalyst activates the hydrophobic alkene **2**, generating an active hydrophobic ruthenocarbene (II—Interfacial reaction in Fig. 4a). This then reacts with hydrophilic alkene **1** at the interface to give a ruthenocycle that collapses to amphiphilic **3**. As the concentration of amphiphilic product **3** increases it self-assembles into micelles (III— Physical autocatalysis in Fig. 4a), and likely incorporates organic material into the micelles, as well as additional activated ruthenocarbenes generated from the decomposition of **3** (IV—Micelle destruction in Fig. 4a)^30^. The productive pathway towards the thermodynamic product **4** will therefore consume **3** (V—Thermodynamic product in Fig. 4a).

This mechanism is supported by the observation that hydrophilic alkene **1**, in the presence of Grubbs 2nd generation catalyst, does not react in water (Supplementary Fig. 15a) to give dimeric **4**, which is the thermodynamic waste product of our system. Amphiphile **3a** in the presence of the metathesis catalyst in water also appears stable and does not react to form **4**. However, if hydrophobic alkene **2a** is added to **3a** in water then it is fully consumed to give **4** (Fig. 4b). This experiment suggests that Grubbs 2nd may not be the catalytic active species responsible for destruction of **3**, but that an activated ruthenocarbene derived from the hydrophobic alkene is involved.

A second set of control reactions was performed under homogeneous conditions using a *t*-BuOH-D_2_O solvent mixture, where all reaction components are soluble. Mixing **1**, **2a**, and catalyst under homogeneous conditions (Fig. 4c) showed rapid formation of **3a** with no lag-period, and the reaction no longer appears autocatalytic. Homodimer **4** is formed in small quantities from the beginning, presumably from the dimerisation of **1**, and the concentration of **4** then slightly increases with a concomitant reduction in the concentration of **3a** (Fig. 4c). The selective and rapid formation of **3a** compared to the slow dimerisation of **1** to form **4** under homogeneous condition is consistent with a study on cross-metathesis methods^32,33^. Formation of **4** from **1** was confirmed by a separate control experiment, in the absence of hydrophobic alkene **2a** (Supplementary Fig. 15b). The incomplete conversion of **3a** to **4** is also observed in a separate homogeneous control reaction (Supplementary Fig. 15c), which is in contrast to the relatively rapid conversion of **3a** to **4** under biphasic conditions (see Figs. 2a or 4b).

While phase separation is a requirement for physical autocatalysis, why biphasic conditions are necessary for replicator destruction is less obvious, and both kinetic and thermodynamic factors may be at play. First, from the perspective of **3a**, higher local concentrations of ruthenocarbenes in micelles of **3a** would be expected than under homogeneous conditions, and this would facilitate the destruction of **3a**. The effective equilibrium position may be altered between phase separated and homogenous conditions. For example, on destruction of **3a** to form **4** and **2a**, via **5**, the phase separation of the extruded products might push the equilibrium towards increased consumption of **3a** (Fig. 4). What is clear is that the phase separation both allows for micelle-mediated autocatalysis to occur and for the productive consumption of the self-replicator.

In conclusion, we have designed and operated a small molecule self-replicator whose formation is triggered by a physical autocatalytic reaction across a phase separation. In closed systems the maximum population of the self-assembled replicator is formed following a non-linear, far-from-equilibrium regime. The metastable self-assembled replicator is concurrently consumed, becomes depleted and moves towards thermodynamic equilibrium with the formation of a waste product. Addition of chemical fuel allows high replicator populations to be restored before the inevitable move toward equilibrium. Because formation and destruction of the self-replicator occur concurrently, the system is dynamic and allows temporal control of the self-replicator population. These studies demonstrate that for a small molecule self-replicator, a far-from-equilibrium population of replicators can be controlled in a fully synthetic system designed on first principles of chemical reactivity. Overall, the thermodynamic instability of the replicator enables the system to mimic two fundamental properties of living systems—the ability to self-replicate and persist far-from-equilibrium. Many living systems *grosso modo* are replicators working far-from-equilibrium, which are inevitably destroyed unless they are sustained. Study of dynamic metastable replicators may help to understand how to create minimal life in the laboratory, and provide physical models to study these fundamental properties outside of living systems.

## Methods

### General experimental details

Reagents obtained from Sigma-Aldrich, Alfa, Fluorochem and TCI suppliers were used directly as supplied. All anhydrous reactions were carried out in flame-dried glassware and under an inert atmosphere of argon provided by a balloon. All reactions were stirred with magnetic followers. Flash column chromatography was performed using silica gel (60 Å, 0.033–0.070 mm, BDH). TLC analyses were performed on Merck Kiesegel 60 F_254_ 0.25 mm precoated silica plates. See supplementary information for synthetic procedures, including Supplementary Figures 1–10.

### Kinetic analysis

Kinetic analyses were performed using a Waters acquity ultra performance liquid chromatography (UPLC) H-Class system with photodiode array (PDA) detector. Instrument control and data processing were performed using Empower software. Acquity UPLC BEH C18 column, 2.1×50 mm with a 1.7 µm size particle was used. A mixture of MeOH:H_2_O with a gradient of 5:95 → 95:5 over 5 min was used as mobile phase. Calibration showing linear fitting was obtained (Supplementary Figure 11). See supplementary information for more details, including chromatograms (Supplementary Figures 12–13).

### Critical micelle concentration determination

An established fluorimetric method was used for the CMC determination of alkenes **3a** and **3b**^34^. Analyses were performed using an Edinburgh Instruments Spectrofluorometer FS5 model. Instrument control and data processing were performed using Fluoracle software. Measurements were done using an equilibrated heating probe at 60 °C in quartz cuvettes with 3.0 mL of sample solution. Excitation wavelength was 358 nm and emission wavelength was 430 nm as reported in the literature using 1,6-diphenyl-1,3,5-hexatriene (DPH) as fluorescent molecule. The CMC for alkenes **3a** and **3b** can be extracted from the representation of the Emission vs. Concentration (Supplementary Figures 16 and 17).

### Dynamic light scattering

Analyses were performed using a Malvern Zetasizer Nano ZEN5600 model system recording particle and molecule size. Instrument control and data processing were performed using Zetasizer software. Disposable plastic cuvettes were used with 1.0 mL of sample solution. Three repetitions of ten measurements were done for every concentration, starting from 20 mM with subsequent dilutions. Measurements were done using an equilibrated heating probe at 60 °C, setting the appropriate parameters for water (Supplementary Figures 18 and 19).

### TEM experiments

Images were produced using negative staining. An aliquot of 10 µl of sample was applied to freshly glow discharged carbon Formvar 200 mesh copper grids for 2 min, blotted with filter paper and stained with 2% uranyl acetate for 10 s, then blotted and air dried. Grids were imaged in a FEI Tecnai 12 TEM at 120 kV using a Gatan OneView CMOS camera (Supplementary Figures 20 and 21).

### Compounds characterisation

^1^H NMR and ^13^C NMR spectra were recorded on a 400 MHz or 500 MHz spectrometer in CDCl_3_ or CD_3_OD and referenced to residual solvent peaks (Supplementary Figures 22–31). Infrared spectra were recorded as thin films of neat samples on a Bruker Tensor 27 FT-IR spectrometer equipped with Attenuated Total Reflectance sampling accessories. High resolution mass spectra are given to four decimal places and were recorded on a Bruker MicroTof (resolution = 10,000 FWHM) under conditions of electrospray ionisation (ESI), electronic ionisation (EI) or chemical ionisation (CI). Optical rotations were measured at 25 °C using a sodium lamp in the appropriate solvent. Melting points (m.p.) were obtained from recrystallised samples using a Lecia VMTG heated-stage microscope and are uncorrected.

### Data availability

All reported data are available from the authors on request.

## Electronic supplementary material

Supplementary Information
