# Supplementary material for: A transient self-assembling self-replicator
Source: Nat Commun. 2018 Jun 8;9:2239. doi: 10.1038/s41467-018-04670-2 (PMC5993787; doi:10.1038/s41467-018-04670-2)
Supplement: Supplementary file 1 — Supplementary Information [file 41467_2018_4670_MOESM1_ESM.pdf]

Supplementary Information

A Transient

Self-Assembling Self-Replicator

Colomer et al.

Table of Contents

Supplementary methods S3

General experimental details S3

Synthesis and Characterization of chemical compounds S3

General procedure for homogeneous Ru-catalyzed alkene cross-metathesis S3

Synthesis of **2a** S4

Synthesis of **2b** S4

Synthesis of **S1** S5

Synthesis of **S2** S5

Synthesis of **S3a** S6

Synthesis of **S3b** S7

General procedure for acetate deprotection S8

Synthesis of **1** S8

Synthesis of **3a** S9

Synthesis of **3b** S9

General procedure for biphasic Ru-catalyzed alkene cross-metatesis S10

Synthesis of **4** S10

Kinetic analyses S11

Calibration of the detection method S11

Kinetic analyses using hydrophobic alkene **2a** and **2b** S12

Kinetic analyses for the seeding experiment using **3b** S13

Re-fuelling experiment details S13

Control experiments S14

Aggregation properties S14

Critical micelle concentration (CMC) determination S14

Particle size measure using Dynamic Light Scattering (DLS) S16

TEM experiments S18

NMR spectra S20

Supplementary references S30

Supplementary methods

General experimental details

^1H NMR and ^{13}C NMR spectra were recorded on a 400 MHz or 500MHz spectrometer in $CDCl_3$ or CD_3OD and referenced to residual solvent peaks. Chemical shifts are quoted in ppm (parts per million) to the nearest 0.01 ppm with signal splitting recorded as singlet (s), doublet (d), triplet (t), quartet (q), quintet (quint), septet (sept.), multiplet (m) and broad singlet (br. s). Coupling constants, *J*, are measured in Hz to the nearest 0.1 Hz. ^1H NMR and ^{13}C NMR spectra were recorded at room temperature. Infrared spectra were recorded as thin films of neat samples on a Bruker Tensor 27 FT-IR spectrometer equipped with Attenuated Total Reflectance sampling accessories. High resolution mass spectra are given to four decimal places and were recorded on a Bruker MicroTof (resolution = 10000 FWHM) under conditions of electrospray ionization (ESI), electronic ionization (EI) or chemical ionization (CI). Optical rotations were measured at 25 ºC using a sodium lamp in the appropriate solvent. Melting points (m.p.) were obtained from recrystallized samples using a Lecia VMTG heated-stage microscope and are uncorrected. The solvent systems used for recrystallization are quoted in parentheses. Flash column chromatography was performed using silica gel (60 Å, 0.033-0.070 mm, BDH). TLC analyses were performed on Merck Kiesegel 60 F_{254} 0.25 mm precoated silica plates. Reagents obtained from Sigma-Aldrich, Alfa, Fluorochem and TCI suppliers were used directly as supplied. All anhydrous reactions were carried out in flame-dried glassware and under an inert atmosphere of argon provided by a balloon. All reactions were stirred with magnetic followers.

Synthesis and characterization of chemical compounds

General procedure for homogeneous Ru-catalyzed alkene cross-metathesis.

To a flame-dried flask, charged with a catalytic amount of Ru-catalyst, under Ar, at room temperature, was added 4.0 mL/mmol of dry solvent (previously degassed, bubbling Ar over 30 min.). A solution of the mixture of alkenes in dry and degassed solvent (1.0 mL/mmol) was added and the mixture was stirred at the appropriate temperature. The reaction was monitored by TLC until completion, and the solvent was evaporated under reduced pressure to give the corresponding alkene, which was purified by chromatography on silica gel using the appropriate mixture of eluents.

Synthesis of 5-Decene, 2a.

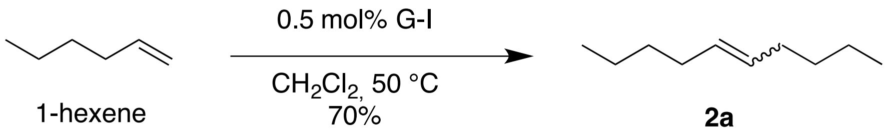

Supplementary Figure 1. **Synthesis of 2a**

From 1-hexene (20.0 mL, 161 mmol) and Grubbs 1st generation catalyst (800 mg, 0.81 mmol), in 50 mL of CH_2Cl_2, following the general procedure, a 4.7:1 *E*:*Z* mixture of alkene **2a** was obtained. Chromatographic purification (pentane) gave a 4.7:1 *E*:*Z* mixture of alkene **2a** (15.7 g, 70%), as a colorless oil. Spectroscopic properties matched those previously reported.[1]

Data for **2a**: $\boldsymbol{R_f}$ 0.80 (pentane). **^{1}H NMR (500 MHz, $CDCl_3$)** δ 5.38-5.41 (2 H, m, C*H*=C*H* *E* isom), 5.35-5.37 (2 H, m, C*H*=C*H* *Z* isom), 2.01-2.06 (4 H, m, 2 × CH_2-CH=CH *Z* isom), 1.96-2.01 (4 H, m, 2 × CH_2-CH=CH *E* isom), 1.29-1.36 (8 H, m, 4 × CH_2), 0.91 (6 H, t, J = 7.1 Hz, 2 × CH_3 *Z* isom), 0.90 (6 H, t, J = 7.2 Hz, 2 × CH_3 *E* isom). **^{13}C NMR (125 MHz, $CDCl_3$)** δ 130.5 (2C, *C*H=*C*H *E* isom), 130.0 (2C, *C*H=*C*H *Z* isom), 32.5 (2C, *C*H_2-CH=CH-*C*H_2 *E* isom), 32.2 (2C, 2 × CH_2 *Z* isom), 32.0 (2C, 2 x CH_2 *E* isom), 27.1 (2C, *C*H_2-CH=CH-*C*H_2 *Z* isom), 22.5 (2C, 2 × CH_2 *Z* isom), 22.4 (2C, 2 × CH_2 *E* isom), 14.2 (2C, 2 × CH_3 *Z* isom), 14.1 (2C , 2 × CH_3 *E* isom). **IR** (film): ν_{max} 2981, 1636, 1367, 1152, 954 cm^{-1}. **HRMS** (EI): calculated for $C_{10}H_{20}$ $[M]^+$ requires *m*/*z* 140.1560, found *m*/*z* 140.1556.

Synthesis of 11-Docosene, 2b

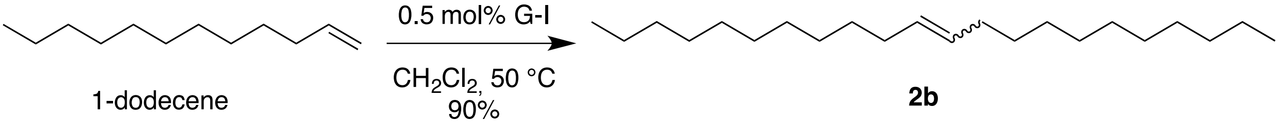

Supplementary Figure 2. **Synthesis of 2b**

From 1-hexene (20 mL, 90 mmol) and Grubbs 1st generation catalyst (370 mg, 0.45 mmol), in 50 mL of CH_2Cl_2, following the general procedure, a 4.2:1 *E*:*Z* mixture of alkene **2b** was obtained. Chromatographic purification (pentane) gave a 4.2:1 *E*:*Z* mixture of alkene **2b** (24.9 g, 90%), as a colorless oil. Spectroscopic properties matched those previously reported.[2]

Data for **2b**: $\boldsymbol{R_f}$ 0.80 (pentane). **^{1}H NMR (500 MHz, $CDCl_3$)** δ 5.38-5.40 (2 H, m, C*H*=C*H* *E* isom), 5.34-5.37 (2 H, m, C*H*=C*H* *Z* isom), 2.00-2.05 (4 H, m, 2 × CH_2-CH=CH *Z* isom), 1.94-2.00 (4 H, m, 2 × CH_2-CH=CH *E* isom), 1.24-1.36 (32 H, m, 16 x CH_2), 0.89 (6 H, t, J = 6.9 Hz, 2 × CH_3). **^{13}C NMR (125 MHz, $CDCl_3$)** δ 130.5 (2C, *C*H=*C*H *E* isom), 130.1 (2C, *C*H=*C*H *Z* isom), 32.8 (2C, *C*H_2-CH=CH-*C*H_2 *E* isom), 32.1 (2C, 2 × CH_2), 29.9 (4C, 4 × CH_2), 29.8 (2C, 2 × CH_2), 29.7 (2C, 2 × CH_2), 29.6 (2C, 2 × CH_2), 29.4 (2C, 2 × CH_2), 27.4 (2C, *C*H_2-CH=CH-*C*H_2 *Z* isom), 22.9 (2C, 2 × CH_2), 14.3 (2C, 2 × CH_3). **IR** (film): ν_{max} 2956, 2921, 2852, 1464, 966, 720 cm^{-1}. **HRMS** (EI): calculated for $C_{22}H_{44}$ $[M]^+$ requires *m*/*z* 308.3438, found *m*/*z* 308.3440.

Synthesis of S1.

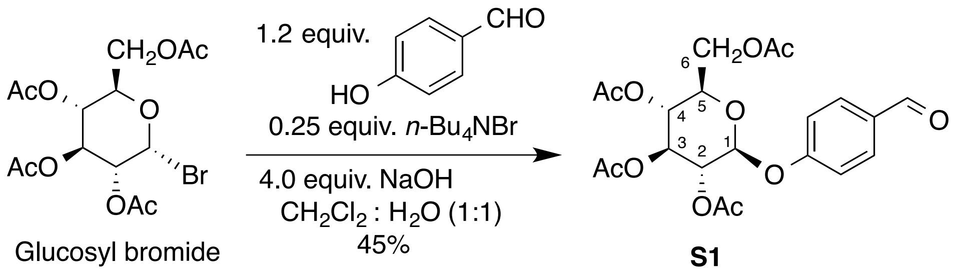

S1

Supplementary Figure 3. **Synthesis of S1**

To a solution of 4-Hydroxybenzaldehyde (1.5 g, 65.7 mmol) in 50 mL of CH_2Cl_2, a solution of NaOH (5.25 g, 131 mmol) and *n*-Bu_4NBr (3.53 g, 11.0 mmol) in 100 mL of H_2O was added. After stirring the solution at rt for 30 min. a solution of 2,3,4,6-tetra-*O*-acetyl-α-D-glucopyranosyl bromide (18.0 g, 43.8 mmol) in 50 mL of CH_2Cl_2 was added and the reaction was stirred vigorously at that temperature until completion (4 days). The reaction was extracted and the organic layer was washed with 5% NaOH (3 x 50 mL). The combined organic layers were dried using Na_2SO_4, filtered and the solvent was evaporated under reduced pressure. Chromatographic purification (gradient elution: 10:90 → 60:40 EtOAc – pentane) gave **S1** (8.9 g, 45%), as a white solid. Spectroscopic properties matched those previously reported.[3]

Data for **S1**: $\boldsymbol{R_f}$ 0.25 (50% EtOAc – pentane). **m.p.** 140.5 °C (10% pentane – Et_2O). $\boldsymbol{[\alpha]^{25}_D}$ = – 28.0 (*c* = 1.50, $CHCl_3$). **^{1}H NMR (500 MHz, $CDCl_3$)** δ 9.92 (1 H, s, CHO), 7.85 (2 H, d, *J* = 8.7 Hz, Ar), 7.10 (2 H, d, *J* = 8.7 Hz, Ar), 5.28-5.34 (2 H, m, 2-H and 3-H), 5.21 (1 H, d, *J* = 7.3 Hz, 1-H), 5.18 (1 H, t, *J* = 9.7 Hz, 4-H), 4.28 (1 H, dd, *J* = 12.3 and 5.5 Hz, 6-H_A), 4.18 (1 H, dd, *J* = 12.3 and 2.4 Hz, 6-H_B), 3.92 (1 H, ddd, *J* = 10.1, 5.5 and 2.4 Hz, 5-H), 2.07 (3 H, s, Me OAc), 2.06 (3 H, s, Me OAc), 2.05 (3 H, s, Me OAc), 2.04 (3 H, s, Me OAc). **^{13}C NMR (125 MHz, $CDCl_3$)** δ 190.8 (CHO), 170.6 (C=O Ac), 170.3 (C=O Ac), 169.5 (C=O Ac), 169.3 (C=O Ac), 161.4 (C Ar), 132.0 (C Ar), 131.9 (2 × CH Ar), 116.9 (2 × CH Ar), 98.2 (C-1), 72.7 (C-2), 72.5 (C-5), 71.1 (C-3), 68.3 (C-4), 62.0 (C-6), 20.8 (Me Ac), 20.75 (Me Ac), 20.74 (Me Ac), 20.72 (Me Ac). **IR** (film): ν_{max} 1749, 1693, 1602, 1368, 1214, 1036, 835, 754 cm^{-1}. **HRMS** (ESI): calculated for $C_{21}H_{24}O_{11}Na$ $[M+Na]^+$ requires *m*/*z* 475.1211, found *m*/*z* 475.1210.

Synthesis of S2.

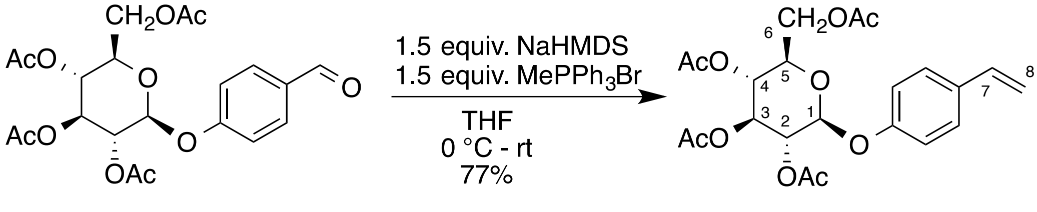

S1 **S2**

Supplementary Figure 4. **Synthesis of S2**

To a cold (0 ºC) suspension of $MePPh_3Br$ (4.9 g, 13.6 mmol) in dry THF, NaHMDS 1.0 M solution in THF (13.6 mL, 13.6 mmol) was added. The suspension turned into a bright yellow solution and was stirred at that temperature for 30 min. A solution of aldehyde **S1** (4.1 g, 9.06 mmol) in 90 mL of dry THF was added and the reaction was warmed to rt and stirred for 12h until completion. The reaction mixture was quenched with saturated NH_4Cl solution (50 mL) and extracted with EtOAc (3 x 100 mL). The combined organic layers were dried using Na_2SO_4, filtered, and the solvent was evaporated under reduced pressure. Chromatographic purification (gradient elution: 10:90 → 60:40 EtOAc – pentane) gave **S2** (3.1 g, 77%), as a white solid.

Data for **S2**: ***R**$_f$* 0.40 (50% EtOAc – pentane). **m.p.** 112.0 °C (10% pentane – Et_2O). **[α]$^{25}_{D}$** = – 22.5 (*c* = 1.20, $CHCl_3$). **^{1}H NMR (500 MHz, $CDCl_3$)** δ 7.34 (2 H, d, *J* = 8.6 Hz, Ar), 6.95 (2 H, d, *J* = 8.8 Hz, Ar), 6.66 (1 H, dd, *J* = 17.6 and 10.9 Hz, 7-H), 5.64 (1 H, dd, *J* = 17.7 and 0.9 Hz, 8-H_{trans}), 5.24-5.32 (2 H, m, 2-H and 3-H), 5.19 (1 H, d, *J* = 10.7 Hz, 8-H_{cis}), 5.17 (1 H, t, *J* = 9.6 Hz, 4-H), 5.08 (1 H, d, *J* = 7.5 Hz, 1-H), 4.29 (1 H, dd, *J* = 12.3 and 5.3 Hz, 6-H_A), 4.17 (1 H, dd, *J* = 12.3 and 2.5 Hz, 6-H_B), 3.86 (1 H, ddd, *J* = 10.1, 5.3 and 2.5 Hz, 5-H), 2.08 (3 H, s, Me OAc), 2.06 (3 H, s, Me OAc), 2.05 (3 H, s, Me OAc), 2.04 (3 H, s, Me OAc). **^{13}C NMR (125 MHz, $CDCl_3$)** δ 170.7 (C=O Ac), 170.4 (C=O Ac), 169.5 (C=O Ac), 169.4 (C=O Ac), 156.6 (C Ar), 136.0 (C-7), 133.2 (C Ar), 127.5 (2 × CH Ar), 117.2 (2 × CH Ar), 113.2 (C-8), 99.3 (C-1), 72.9 (C-2), 72.2 (C-5), 71.3 (C-3), 68.4 (C-4), 62.1 (C-6), 20.9 (Me Ac), 20.80 (Me Ac), 20.78 (Me Ac), 20.75 (Me Ac). **IR** (film): ν_{max} 1748, 1509, 1368, 1220, 1037, 908, 840 cm^{-1}. **HRMS** (ESI): calculated for $C_{22}H_{26}O_{10}Na$ $[M+Na]^+$ requires *m*/*z* 473.1418, found *m*/*z* 473.1413.

Synthesis of S3a.

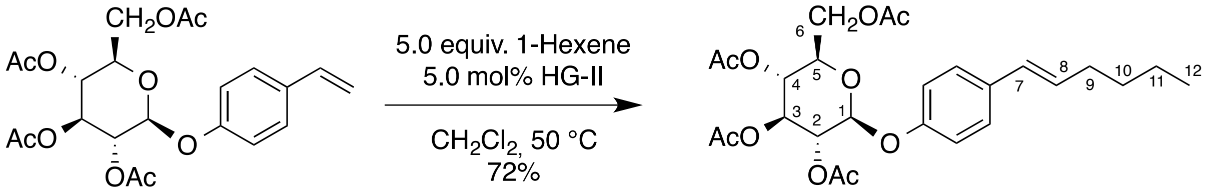

S2 **S3a**

Supplementary Figure 5. **Synthesis of S3a**

From alkene **S2** (300 mg, 0.67 mmol), 1-Hexene (0.4 mL, 3.33 mmol) and Hoveyda-Grubbs 2nd generation catalyst (20 mg, 0.03 mmol), in 7 mL of CH_2Cl_2, following the general procedure, alkene **S3a** was obtained. Chromatographic purification (gradient elution: 10:90 → 50:50 EtOAc – pentane) gave **S3a** (245 mg, 72%), as a white solid.

Data for **S3a**: ***R**$_f$* 0.50 (50% EtOAc – pentane). **m.p.** 136.0 °C (10% pentane – Et_2O). **[α]$^{25}_{D}$** = – 9.4 (*c* = 1.60, $CHCl_3$). **^{1}H NMR (500 MHz, $CDCl_3$)** δ 7.26 (2 H, d, *J* = 8.6 Hz, Ar), 6.91 (2 H, d, *J*

= 8.6 Hz, Ar), 6.32 (1 H, d, *J* = 15.7 Hz, 7-H), 6.12 (1 H, dt, *J* = 15.7 and 6.9 Hz, 8-H), 5.24-5.32 (2 H, m, 2-H and 3-H), 5.16 (1 H, t, *J* = 9.3 Hz, 4-H), 5.05 (1 H, d, *J* = 7.2 Hz, 1-H), 4.30 (1 H, dd, *J* = 12.3 and 5.3 Hz, 6-H_A), 4.16 (1 H, dd, *J* = 12.2 and 2.4 Hz, 6-H_B), 3.84 (1 H, ddd, *J* = 9.9 and 5.3 and 2.4 Hz, 5-H), 2.19 (2 H, q, *J* = 6.5 Hz, 9-H_2), 2.08 (3 H, s, Me OAc), 2.06 (3 H, s, Me OAc), 2.04 (3 H, s, Me OAc), 2.03 (3 H, s, Me OAc), 1.43 (2 H, quint, *J* = 7.1 Hz, 10-H_2), 1.36 (2 H, quint, *J* = 7.1 Hz, 11-H_2), 0.92 (3 H, t, *J* = 7.2 Hz, 12-H_3). **^{13}C NMR (125 MHz, $CDCl_3$)** δ 170.6 (C=O Ac), 170.3 (C=O Ac), 169.4 (C=O Ac), 169.3 (C=O Ac), 155.8 (C Ar), 133.5 (C Ar), 130.5 (C-8), 128.7 (C-7), 127.0 (2 × CH Ar), 117.1 (2 × CH Ar), 99.3 (C-1), 72.8 (C-2), 72.0 (C-5), 71.2 (C-3), 68.3 (C-4), 62.0 (C-6), 32.7 (C-9), 31.6 (C-10), 22.3 (C-11), 20.72 (Me Ac), 20.65 (Me Ac), 20.64 (Me Ac), 20.61 (Me Ac), 14.0 (C-12). **IR** (film): ν_{max} 2360, 2341, 1749, 1607, 1509, 1368, 1225, 1043, 966 cm^{-1}. **HRMS** (ESI): calculated for $C_{26}H_{34}O_{10}Na$ $[M+Na]^+$ requires *m/z* 529.2044, found *m/z* 529.2039.

Synthesis of S3b.

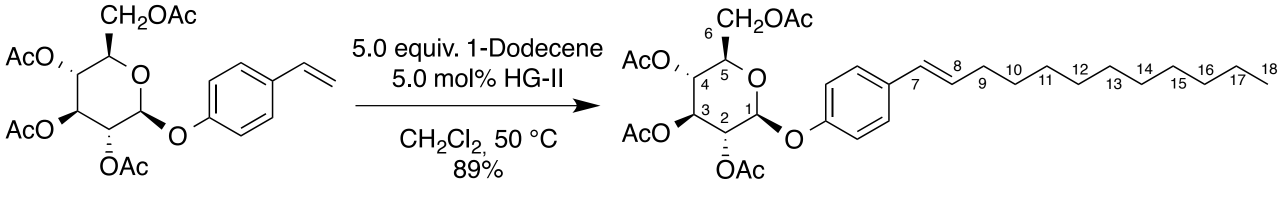

S2 **S3b**

Supplementary Figure 6. **Synthesis of S3b**

From alkene **S2** (300 mg, 0.67 mmol), 1-Dodecene (0.74 mL, 3.3 mmol) and Hoveyda-Grubbs 2nd generation catalyst (20 mg, 0.03 mmol), in 7 mL of CH_2Cl_2, following the general procedure, alkene **S3b** was obtained. Chromatographic purification (gradient elution: 10:90 → 50:50 EtOAc – pentane) gave **S3b** (350 mg, 89%), as a white solid.

Data for **S3b**: ***R_f*** 0.50 (50% EtOAc – pentane). **m.p.** 116.0 °C (10% pentane – Et_2O). **$[\alpha]^{25}_D$** = –7.6 (*c* = 5.01, $CHCl_3$). **^{1}H NMR (500 MHz, $CDCl_3$)** δ 7.25 (2 H, d, *J* = 8.7 Hz, Ar), 6.91 (2 H, d, *J* = 8.6 Hz, Ar), 6.30 (1 H, d, *J* = 15.8 Hz, 7-H), 6.11 (1 H, dt, *J* = 15.8 and 6.9 Hz, 8-H), 5.23-5.31 (2 H, m, 2-H and 3-H), 5.16 (1 H, t, *J* = 9.3 Hz, 4-H), 5.04 (1 H, d, *J* = 7.3 Hz, 1-H), 4.28 (1 H, dd, *J* = 12.3 and 5.3 Hz, 6-H_A), 4.16 (1 H, dd, *J* = 12.3 and 2.4 Hz, 6-H_B), 3.84 (1 H, ddd, *J* = 10.1, 5.3 and 2.4 Hz, 5-H), 2.17 (2 H, q, *J* = 7.0 Hz, 9-H_2), 2.07 (3 H, s, Me OAc), 2.05 (3 H, s, Me OAc), 2.04 (3 H, s, Me OAc), 2.02 (3 H, s, Me OAc), 1.44 (2 H, quint, *J* = 7.1 Hz, 10-H_2), 1.22-1.36 (14 H, m, 11-H_2 and 12-H_2 and 13-H_2 and 14-H_2 and 15-H_2 and 16-H_2 and 17-H_2), 0.87 (3 H, t, *J* = 6.7 Hz, 18-H_3). **^{13}C NMR (125 MHz, $CDCl_3$)** δ 170.7 (C=O Ac), 170.3 (C=O Ac), 169.5 (C=O Ac), 169.4 (C=O Ac), 155.9 (C Ar), 133.6 (C Ar), 130.6 (C-7), 128.7 (C-8), 127.0 (2 × CH Ar), 117.2 (2 × CH Ar),

99.4 (C-1), 72.8 (C-2), 72.1 (C-5), 71.3 (C-3), 68.4 (C-4), 62.1 (C-6), 33.1 (C-9), 32.0 and 29.71 and 29.70 and 29.6 and 29.5 and 29.4 and 29.3 and 22.8 (C-10 and C-11 and C-12 and C-13 and C-14 and C-15 and C-16 and C-17), 20.78 (Me Ac), 20.72 (Me Ac), 20.70 (Me Ac), 20.68 (Me Ac), 14.2 (C-18). **IR** (film): ν_{max} 2921, 2851, 1743, 1607, 1509, 1367, 1219, 1043, 909 cm^{-1}. **HRMS** (ESI): calculated for $C_{32}H_{46}O_{10}Na$ $[M+Na]^+$ requires *m/z* 613.2983, found *m/z* 613.2979.

General procedure for acetate deprotection

To a solution of acetate in dry MeOH (10.0 mL/mmol), NaOMe (0.1 equiv) was added in one portion at room temperature. The reaction was monitored by TLC until completion, and quenched with Amberlyst-15, to reach a neutral pH. The mixture was filtered and the solvent was evaporated under reduced pressure to give the corresponding free carbohydrate, which was purified by chromatography on silica gel using the appropriate mixture of eluents.

Synthesis of 1.

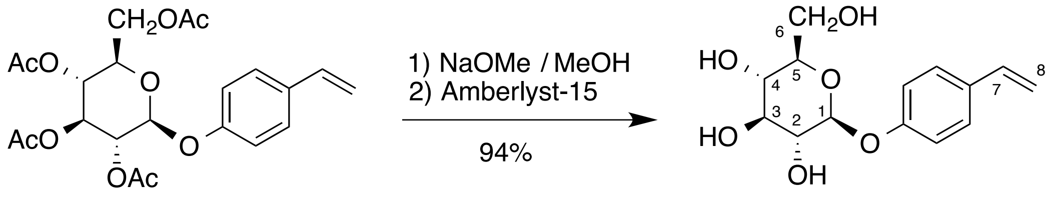

S2 **1**

Supplementary Figure 7. **Synthesis of 1**

From alkene **S2** (2.0 g, 4.4 mmol) and NaOMe (47 mg, 0.80 mmol), in 20.0 mL of MeOH, following the general procedure, alkene **1** was obtained. Chromatographic purification (gradient elution: 5:95 → 25:75 MeOH – CH_2Cl_2) gave **1** (1.24 g, 94%), as a white solid.

Data for **1**: R_f 0.30 (20% MeOH – CH_2Cl_2). **m.p.** 187.0 °C (10% Et_2O – CH_2Cl_2). $[\alpha]^{25}_D$ = –57.5 (*c* = 2.01, MeOH). **^{1}H NMR (500 MHz, CD_3OD)** δ 7.36 (2 H, d, *J* = 8.7 Hz, Ar), 7.05 (2 H, d, *J* = 8.7 Hz, Ar), 6.67 (1 H, dd, *J* = 17.6 and 10.9 Hz, 7-H), 5.64 (1 H, dd, *J* = 17.6 and 1.0 Hz, 8-H_{trans}), 5.12 (1 H, dd, *J* = 10.9 and 1.0 Hz, 8-H_{cis}), 4.91 (1 H, d, *J* = 7.3 Hz, 1-H), 3.90 (1 H, dd, *J* = 12.1 and 2.2 Hz, 6-H_A), 3.70 (1 H, dd, *J* = 12.0 and 5.5 Hz, 6-H_B), 3.37-3.48 (4 H, m, 2-H, 3-H, 4-H and 5-H). **^{13}C NMR (125 MHz, CD_3OD)** δ 158.8 (C Ar), 137.5 (C-7), 133.4 (C Ar), 128.3 (2 × CH Ar), 117.7 (2 × CH Ar), 112.4 (C-8), 102.2 (C-1), 78.1 (C-2), 78.0 (C-5), 74.9 (C-3), 71.4 (C-4), 62.5 (C-6). **IR** (film): ν_{max} 3245, 2950, 2921, 2890, 2850, 1626, 1606, 1508, 1367, 1241, 1114, 1075, 1045, 1018, 909, 837 cm^{-1}. **HRMS** (ESI): calculated for $C_{14}H_{18}O_6Na$ $[M+Na]^+$ requires *m/z* 305.0996, found *m/z* 305.0997.

Synthesis of 3a.

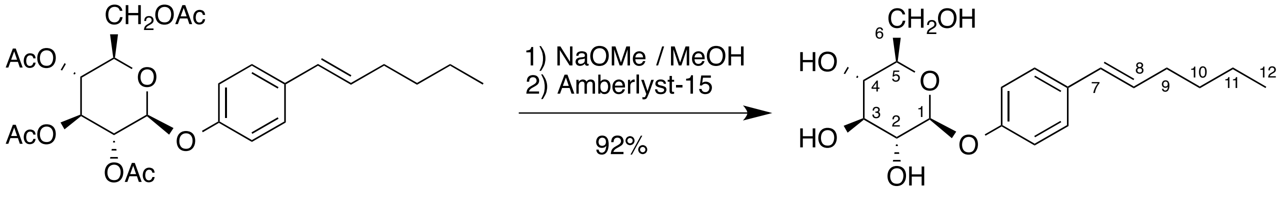

S3a **3a**

Supplementary Figure 8. **Synthesis of 3a**

From alkene **S3a** (350 mg, 0.69 mmol) and NaOMe (7 mg, 0.14 mmol), in 5.0 mL of MeOH, following the general procedure, alkene **3a** was obtained. Chromatographic purification (gradient elution: 10:90 → 50:50 MeOH – CH_2Cl_2) gave **S3a** (233 mg, 92%), as a white solid.

Data for **S3a**: ***R_f*** 0.35 (20% MeOH – CH_2Cl_2). **m.p.** 127.0 °C (10% Et_2O – CH_2Cl_2). **$[\alpha]^{25}_D$** = – 49.4 (*c* = 1.60, MeOH). **^{1}H NMR (500 MHz, CD_3OD)** δ 7.27 (2 H, d, *J* = 8.7 Hz, Ar), 7.02 (2 H, d, *J* = 8.7 Hz, Ar), 6.32 (1 H, d, *J* = 15.8 Hz, 7-H), 6.12 (1 H, dt, *J* = 15.7 and 6.9 Hz, 8-H), 4.88 (1 H, d, *J* = 7.3 Hz, 1-H), 3.89 (1 H, dd, *J* = 12.0 and 2.2 Hz, 6-H_A), 3.70 (1 H, dd, *J* = 12.1 and 5.3 Hz, 6-H_B), 3.37-3.47 (4 H, m, 2-H and 3-H and 4-H and 5-H), 2.14-2.22 (2 H, m, 9-H_2), 1.42-1.53 (2 H, m, 10-H_2), 1.33-1.42 (2 H, m, 11-H_2), 0.94 (3 H, t, *J* = 7.2 Hz, 12-H_3). **^{13}C NMR (125 MHz, CD_3OD)** δ 158.1 (C Ar), 133.8 (C Ar), 130.4 (C-7), 130.3 (C-8), 127.9 (2 × CH Ar), 117.7 (2 × CH Ar), 102.3 (C-1), 78.1 (C-2), 78.0 (C-5), 74.9 (C-3), 71.4 (C-4), 62.5 (C-6), 33.8 (C-9), 32.9 (C-10), 23.3 (C-11), 14.3 (C-12). **IR** (film): ν_{max} 3364, 2959, 2926, 1509, 1239, 1104, 1073, 1046, 1018, 961 cm^{-1}. **HRMS** (ESI): calculated for $C_{18}H_{26}O_6Na$ $[M+Na]^+$ requires *m*/*z* 361.1622, found *m*/*z* 361.1622.

Synthesis of 3b.

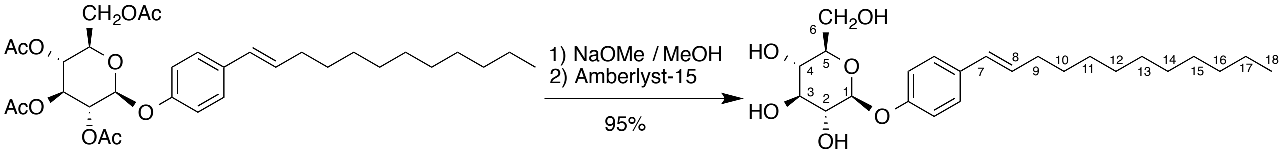

S3b **3b**

Supplementary Figure 9. **Synthesis of 3b**

From alkene **S3b** (280 g, 0.47 mmol) and NaOMe (5 mg, 0.09 mmol), in 5.0 mL of MeOH, following the general procedure, alkene **3b** was obtained. Chromatographic purification (gradient elution: 10:90 → 50:50 MeOH – CH_2Cl_2) gave **3b** (1888 g, 95%), as a white solid.

Data for **3b**: ***R_f*** 0.40 (20% MeOH – CH_2Cl_2). **m.p.** 117.0 °C (10% Et_2O – CH_2Cl_2). **$[\alpha]^{25}_D$** = – 38.0 (*c* = 5.00, MeOH). **^{1}H NMR (500 MHz, CD_3OD)** δ 7.25 (2 H, d, *J* = 8.7 Hz, Ar), 7.01 (2 H, d, *J* = 8.8 Hz, Ar), 6.31 (1 H, d, *J* = 15.8 Hz, 7-H), 6.11 (1 H, dt, *J* = 15.7 and 6.9 Hz, 8-H), 4.87 (1 H, d, *J* = 7.5 Hz, 1-H), 3.88 (1 H, dd, *J* = 12.1 and 2.1 Hz, 6-H_A), 3.70 (1 H, dd, *J* = 12.0 and 5.1 Hz, 6-H_B), 3.38-3.47 (4 H, m, 2-H and 3-H and 4-H and 5-H), 2.17 (2 H, q, *J* = 7.0 Hz, 9-H_2), 1.45 (2 H,

quint, $J = 7.2$ Hz, 10-H_2), 1.23-1.36 (14 H, m, 11-H_2 and 12-H_2 and 13-H_2 and 14-H_2 and 15-H_2 and 16-H_2 and 17-H_2), 0.89 (3 H, t, $J = 6.9$ Hz, 18-H_3). **^{13}C NMR (125 MHz, CD_3OD)** δ 158.1 (C Ar), 133.7 (C Ar), 130.4 (C-7), 130.3 (C-8), 127.9 (2 × CH Ar), 117.7 (2 × CH Ar), 102.3 (C-1), 78.1 (C-2), 77.9 (C-5), 74.9 (C-3), 71.3 (C-4), 62.5 (C-6), 34.1 (C-9), 33.1 and 30.74 and 30.73 and 30.64 and 30.62 and 30.45 and 30.34 and 23.7 (C-10 and C-11 and C-12 and C-13 and C-14 and C-15 and C-16 and C-17), 14.5 (C-18). **IR** (film): ν_{max} 3346, 2981, 2920, 1636, 1458, 1382, 1238, 1071, 1017, 958 cm^{-1}. **HRMS** (ESI): calculated for $C_{24}H_{38}O_6Na$ $[M+Na]^+$ requires *m*/*z* 445.2561, found *m*/*z* 445.2565.

General procedure for biphasic Ru-catalyzed alkene cross-metatesis

To a flame-dried flask, charged with a catalytic amount of Ru-catalyst, under Ar, at room temperature, was added a solution of hydrophilic alkene in dry D_2O (previously degassed, bubbling Ar over 30 min.). Hydrophobic alkene was added and the mixture was stirred at the appropriate temperature and stirring speed. The reaction was monitored using Ultra Performance Liquid Chromatography (UPLC) until completion. The solvent was evaporated under reduced pressure to give the corresponding product that was purified by chromatography on silica gel using the appropriate mixture of eluents.

Synthesis of 4.

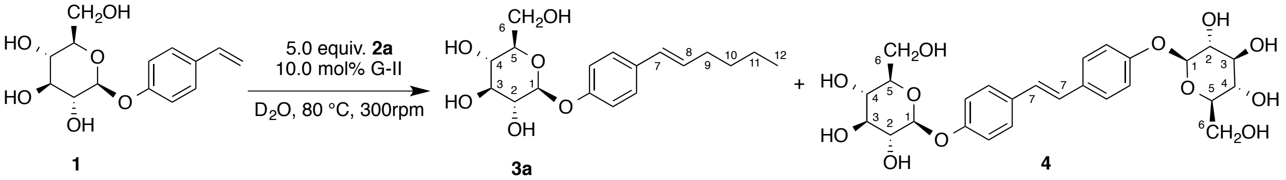

Supplementary Figure 10. **Synthesis of 4**

From alkenes **1** (30 mg, 0.106 mmol) and **2a** (0.20 mL, 0.53 mmol) and Grubbs 2nd generation catalyst (10 mg, 0.01 mmol), in 1.5 mL of D_2O (70 mM), following the general procedure at 80 °C and 300 rpm, alkenes **3a** and **4** were obtained.

Stopping the reaction after 4h, after chromatographic purification (gradient elution: 10:90 → 50:50 MeOH – CH_2Cl_2) gave **3a** (25 mg, 70%) and **4** (4 mg, 18%), as white solids.

Stopping the reaction after 24h, after chromatographic purification (gradient elution: 10:90 → 50:50 MeOH – CH_2Cl_2) gave **4** (17 mg, 59%), as white solid.

Data for **4**: ***R_f*** 0.50 (50% MeOH – CH_2Cl_2). **m.p.** 245.0 °C (10% MeOH – CH_2Cl_2). **$[\alpha]^{25}_D$** = – 71.4 (*c* = 1.40, H_2O). **^{1}H NMR (500 MHz, CD_3OD)** δ 7.46 (4 H, d, $J = 8.8$ Hz, Ar), 7.08 (4 H, d, $J = 8.8$ Hz, Ar), 7.00 (2 H, s, 7-H), 4.92 (2 H, t, $J = 7.6$ Hz, 1-H), 3.91 (2 H, dd, $J = 12.1$ and 2.2 Hz, 6-H_A), 3.71 (2 H, dd, $J = 12.1$ and 5.5 Hz, 6-H_B), 3.44-3.48 (6 H, m, 2-H, 3-H and 5-H), 3.38-3.42 (2 H, m, 4-H). **^{13}C NMR (125 MHz, CD_3OD)** δ 158.5 (2C, 2 × C Ar), 133.5 (2C, 2 × C Ar), 128.4 (4C,

4 × CH Ar), 127.7 (C-7), 117.9 (4C, 4 × CH Ar), 102.3 (C-1), 78.2 (C-5), 78.0 (C-2), 74.9 (C-3), 71.4 (C-4), 62.5 (C-6). **IR** (film): ν_{max} 3337, 2981, 1626, 1580, 670 cm^{-1}. **HRMS** (ESI): calculated for $C_{26}H_{32}O_{12}Na$ [M+Na]$^+$ requires *m/z* 559.1786, found *m/z* 559.1786.

Kinetic analysis

Kinetic analyses were performed using a Waters Acquity Ultra Performance Liquid Chromatography (UPLC) H-Class system with Photodiode Array (PDA) detector. Instrument control and data processing were performed using Empower software. Acquity UPLC BEH C18 coloumn, 2.1 x 50 mm with a 1.7 μm size particle was used. A mixture of H_2O:MeOH with a gradient of 5:95 → 95:5 over 5 min was used as mobile phase.

Quantitative results were obtained using a 0.5mM solution of Phloroglucinol in H_2O as standard. The reactions were monitored extracting an aliquot of 20 μL at each time point (usually every 30min or every 1h), diluting to 1.0mL using a 0.5mM solution of standard. We opted to used 214nm wavelength to analyse the data, where all the species, including the standard, have a local maximum of absorbance.

Calibration of the detection method

Calibration showing linear fitting was obtained for both systems, involving alkenes **3a** and **3b**. The calibration was made using solutions containing all the specie monitored in the real system, meaning alkene starting material **1**, alkene cross-product **3a** or **3b** and homodimer **4**. Solutions containing a fixed concentration of 0.5mM in standard and four different concentrations (5.0, 2.5, 1.25 and 0.625mM) of the alkenes **1**, **3a** or **3b** and **4** were prepared. The ratio of Absorbance (alkene) / Absorbance (standard) was plotted vs. the ratio of Concentration (alkene) / Concentration (standard). The response factor (Rf) for every alkene was extracted from the linear fitting (Supplementary Figure 11). These results are the average of three repetitions.

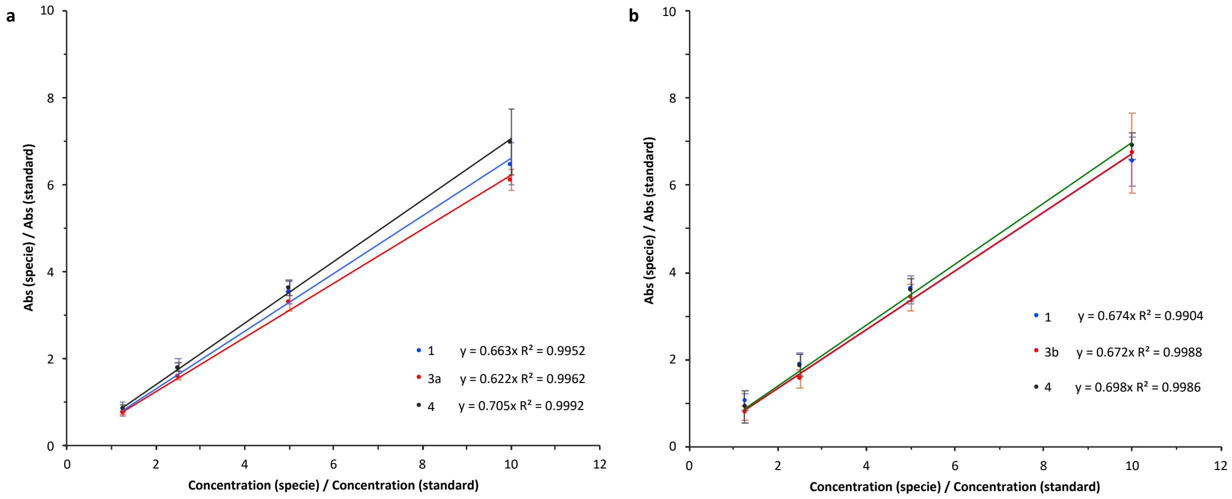

Supplementary Figure 11. **Calibration of the detection method. (a)** For the system involving alkene **3a. (b)** For the system involving **3b**. Error bars represent standard deviation obtained from three repetitions.

Kinetic analyses for 1 (blue), 3 (red) and 4 (black) using hydrophobic alkene 2a and 2b: chromatograms overlapping.

The data used to study the kinetic behaviour shown in Figure 2a-b was extracted from the chromatograms shown below in Supplementary Figures 12 and 13, using alkenes **1** (30 mg, 0.106 mmol) and **2a** (0.20 mL, 0.53 mmol) and Grubbs 2nd generation catalyst (10 mg, 0.01 mmol), in 1.5 mL of D_2O (70 mM), following the general procedure at 80 °C and 300 rpm.

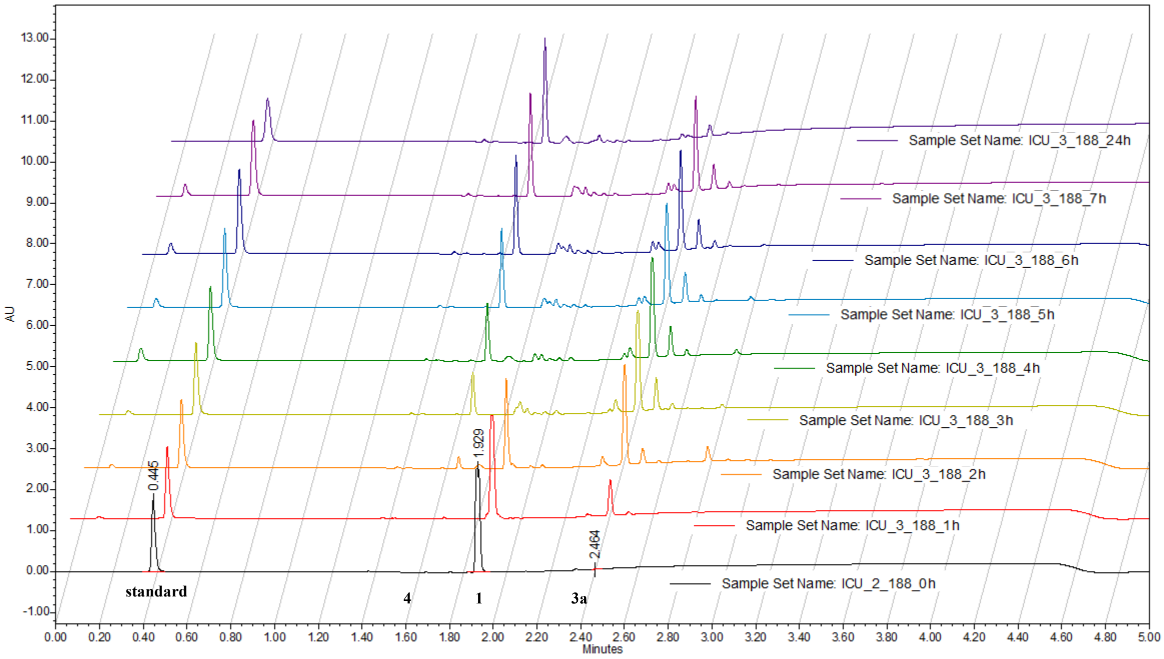

Supplementary Figure 12. Recorded chromatograms at 214nm from 0-24h for alkene **3a** system.

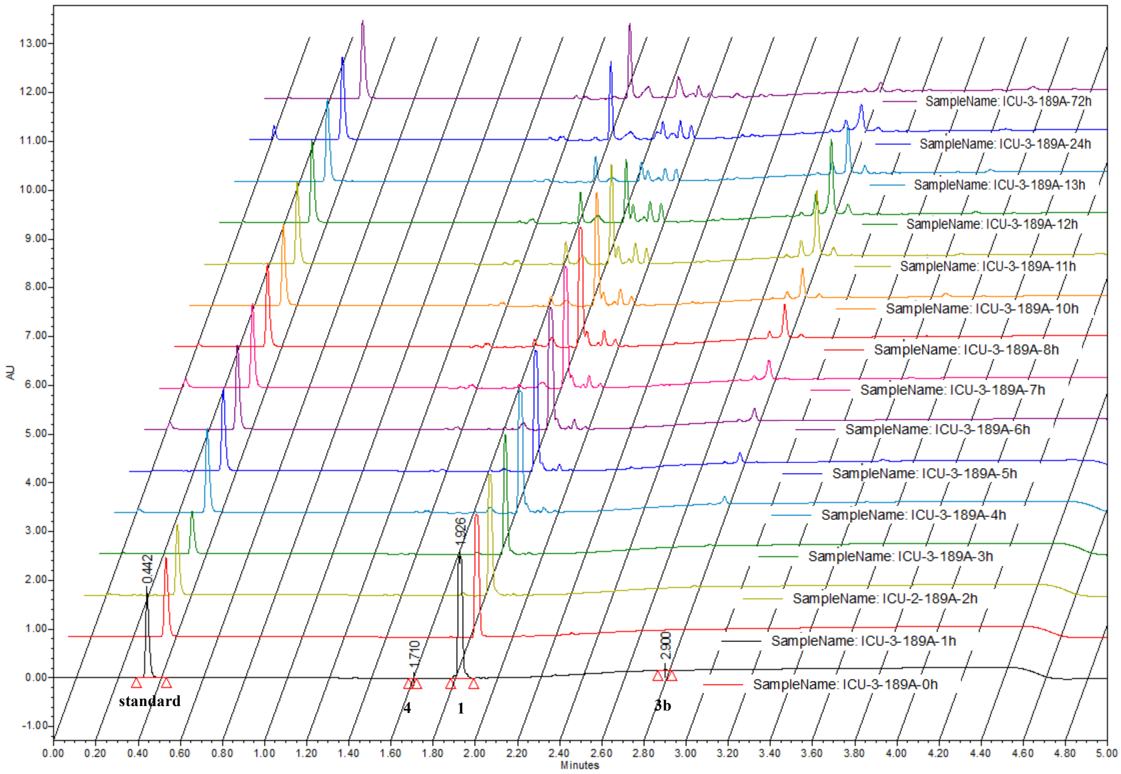

Supplementary Figure 13. Recorded chromatograms at 214nm from 0-24h for alkene **3b** system.

Kinetic analyses for the seeding experiment of the system 2b / 3b with 20 mol% of 3b.

The seeding experiments were performed using alkenes **1** (30 mg, 0.106 mmol) and **2a** (0.20 mL, 0.53 mmol) and Grubbs 2nd generation catalyst (10 mg, 0.01 mmol), in 1.5 mL of D_2O (70 mM), following the general procedure, at 80 °C and 300 rpm, using 20 mol% of amphiphile **3b** present from the beginning of the reaction.

Adding 20 mol% of **3b** from the beginning decreases the initial lag-period in both the starting material hydrophilic alkene **1** consumption (Supplementary Fig. 14a) and the cross-product **3b** formation (Supplementary Fig. 14b).

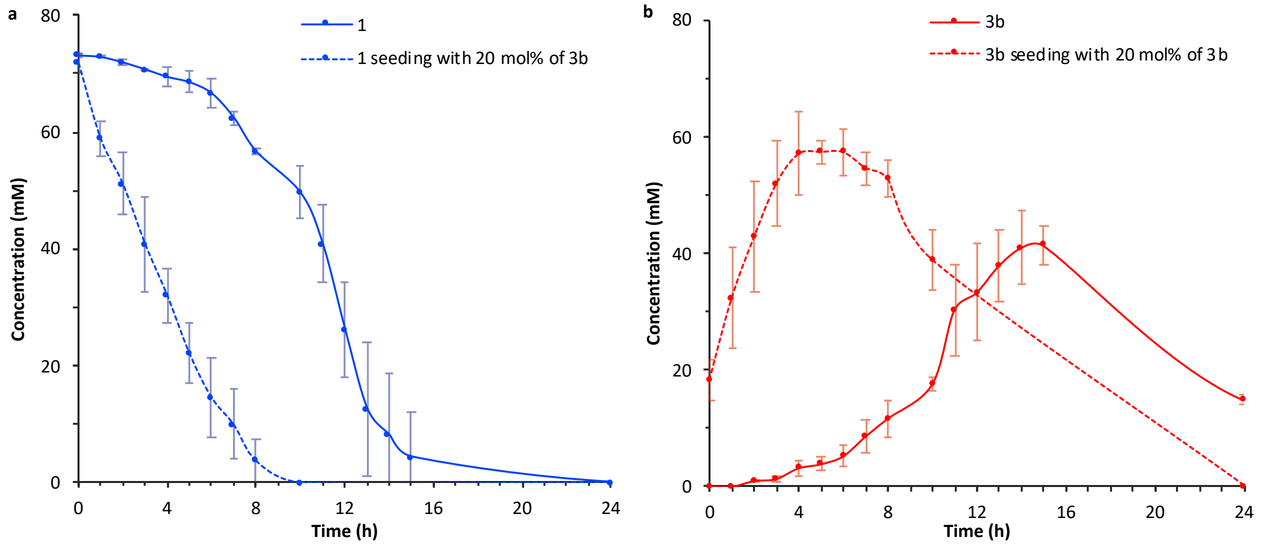

Supplementary Figure 14. **Kinetic analysis representing concentration vs. time, comparing non-seeded reaction (straight line) and seeding the reaction with 20 mol% of 3b (dashed line)**. **(a)** Monitoring starting material **1** consumption and **(b)** Monitoring cross-product **3b** formation. Error bars represent standard deviation obtained from three repetitions.

Re-fuelling experiment details

The re-fuelling experiments were carried out using alkenes **1** (30 mg, 0.106 mmol) and **2a** (0.20 mL, 0.53 mmol) and Grubbs 2nd generation catalyst (10 mg, 0.01 mmol), in 1.5 mL of D_2O (70 mM), following the general procedure at 80 °C and 900 rpm. After 4 hours, when both starting material hydrophilic alkene **1** and amphiphile self-replicator **3a** have been consumed, another set of starting materials **1** (30 mg, 0.106 mmol) and **2a** (0.20 mL, 0.53 mmol) were added. After another 6 hours (10 hours in total from the beginning of the experiment), a second batch of starting materials was re-supplied.

Control experiments

In D_2O no reaction of **1** and Grubbs 2nd to form **4** is observed. Constant concentration of hydrophilic alkene **1** over time is detected (Supplementary Fig. 15a). In contrast, under homogeneous conditions (*t*-BuOH-D_2O) starting material **1** is converted to **4** in the presence of Grubb's 2nd (Supplementary Fig. 15b).

Under homogeneous conditions (*t*-BuOH-D_2O), product **4** is formed in small amounts from **3a** (Supplementary Fig. 15c), but reaction does not go to completion. This is in sharp contrast to the phase separation experiment (see Fig. 4b).

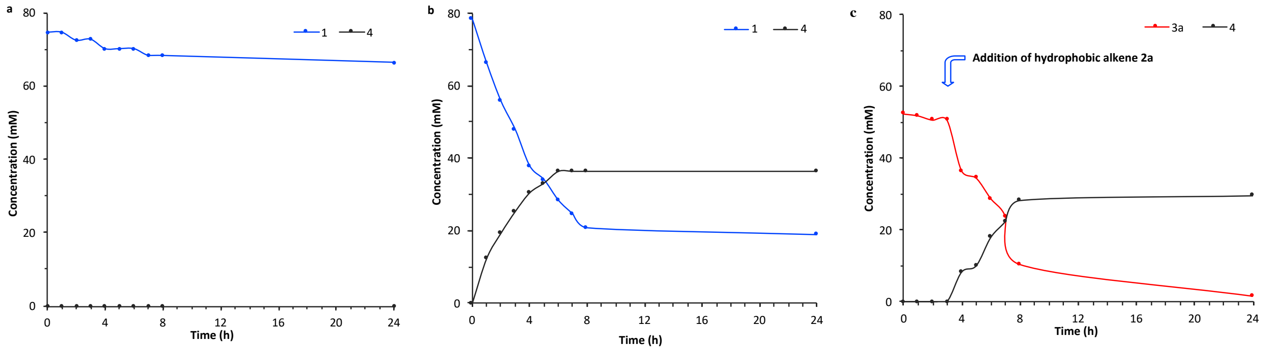

Supplementary Figure 15. Kinetic analysis representing concentration vs. time of 1 (blue), 3 (red) and 4 (black) for the control experiments. (a) Under phase separation using D_2O. **(b) - (c)** Under homogeneous conditions, using *t*-BuOH-D_2O.

Aggregation properties

Two different sets of experiments were performed to determine the aggregation properties of alkenes **3a** and **3b** in water solution.

Critical micelle concentration (CMC) determination

An established fluorimetric method reported in the literature[4] was used for the CMC determination of alkenes **3a** and **3b**. Analyses were performed using an Edinburgh Instruments Spectrofluorometer FS5 model. Instrument control and data processing were performed using Fluoracle software. Measurements were done using an equilibrated heating probe at 60 °C in quartz cuvettes with 3.0 mL of sample solution. Excitation wavelength was 358nm and emission wavelength was 430nm as reported in the literature using 1,6-diphenyl-1,3,5-hexatriene (DPH) as fluorescent molecule. From the representation of the Emission vs. Concentration the CMC can be extracted for alkenes **3a** (Supplementary Figure 16) and **3b** (Supplementary Figure 17).

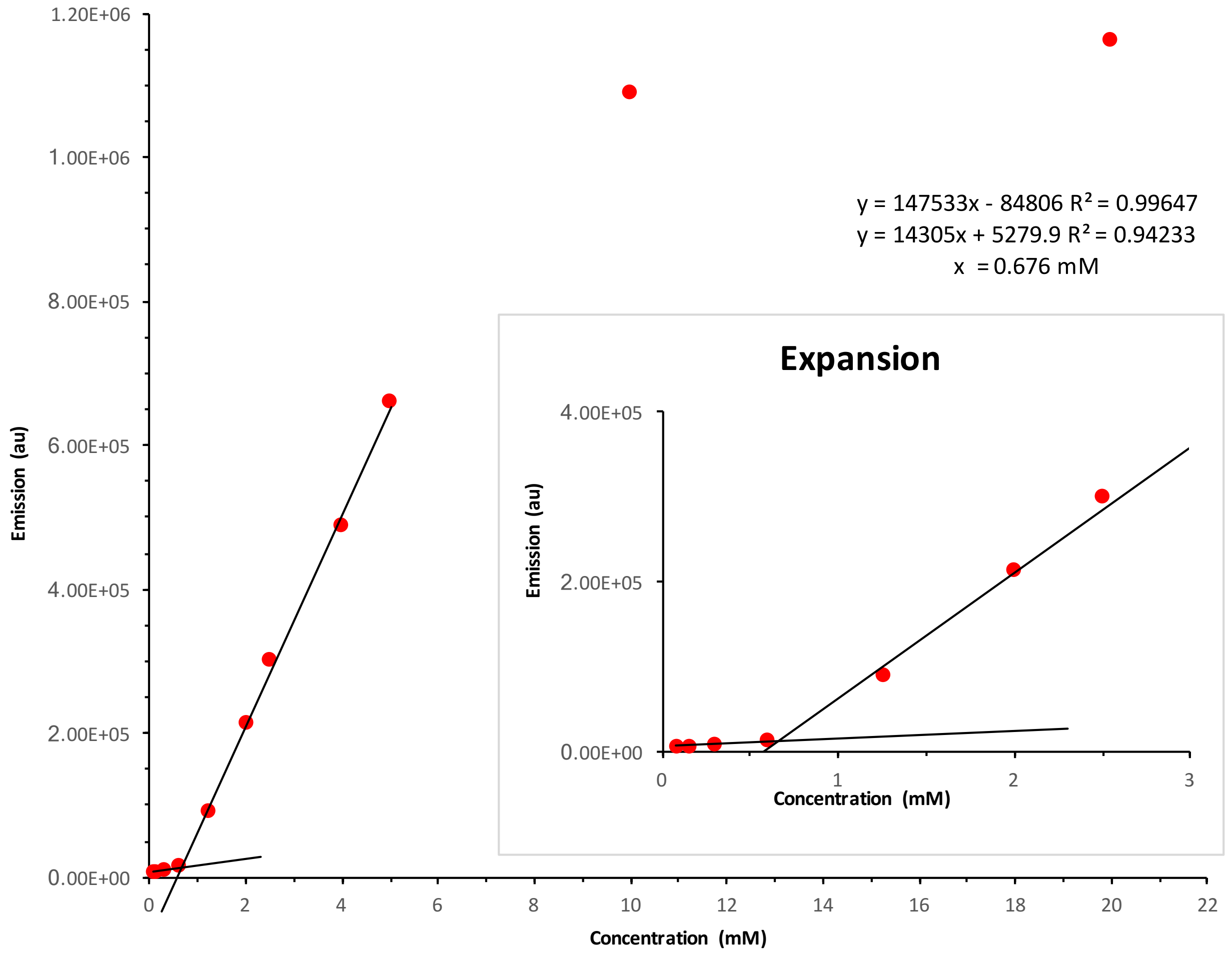

Supplementary Figure 16. Emission vs. concentration and CMC determination for **3a**.

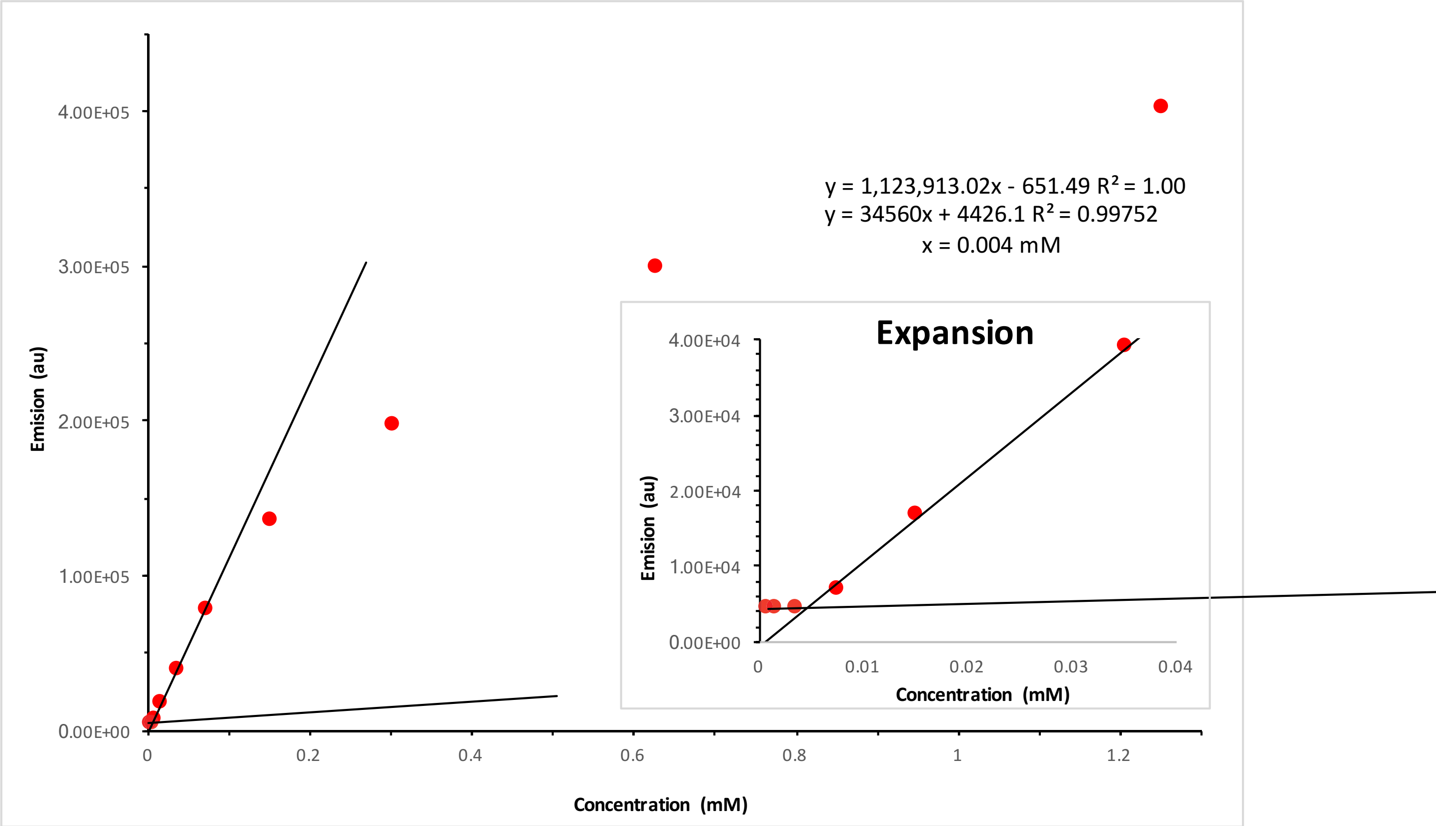

Supplementary Figure 17. Emission vs. concentration and CMC determination for **3b**.

Particle size measure using Dynamic Light Scattering (DLS)

Analyses were performed using a Malvern Zetasizer Nano ZEN5600 model system recording particle and molecule size. Instrument control and data processing were performed using Zetasizer software. Disposable plastic cuvettes were used with 1.0 mL of sample solution. Three repetitions of ten measurements were done for every concentration, starting from 20mM with subsequent dilutions. Measurements were done using an equilibrated heating probe at 60 °C, setting the following parameters for water:

· Dielectric constant = 66.74 · Refractive index = 1.327 · Viscosity = 0.466 · 10^{-3} m · Pa / s

For alkene **3a** a reproducible, consistent and monodisperse size of particle of 28-30nm is detected from 7.5mM to 2.5mM (Supplementary Figures 18a and 18b). At 2.5mM polydispersion is observed (Supplementary Figure 18c). However below 0.625mM micelles of that size cannot be detected anymore (Supplementary Figure 18d), what roughly matched with the CMC = 0.676mM determined for alkene **3a**.

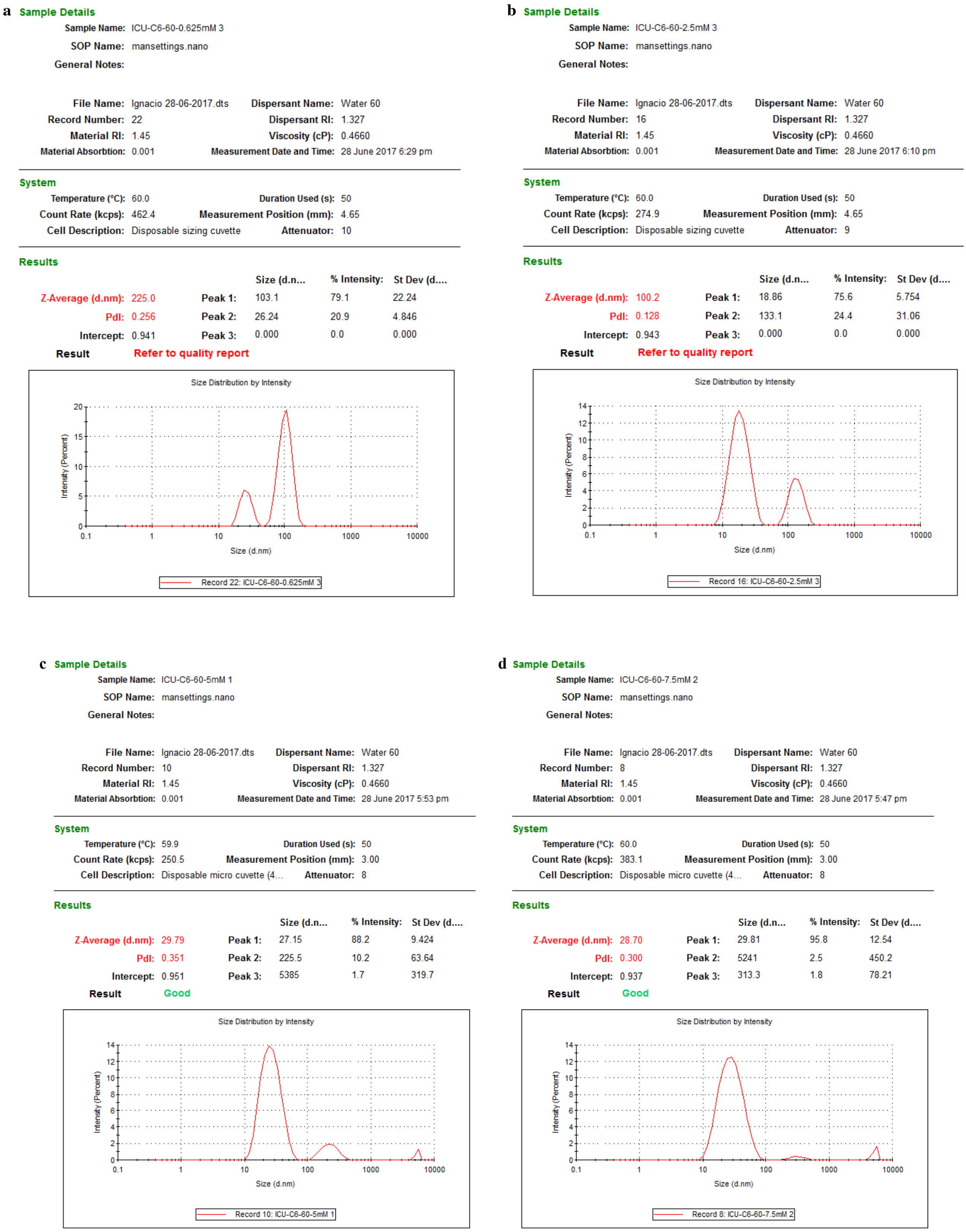

Supplementary Figure 18. DLS experiments using alkene 3a (a) At 7.5mM, **(b)** At 5mM, **(c)** At 2.5mM and **(d)** At 0.625mM

For amphiphile **3b**, at high concentrations, from 10.0mM to 0.3mM, larger particles of 180nm were revealed (Supplementary Figures 19a-b). At lower concentrations, below 0.3mM, particles with an average diameter of 30nm could be detected.

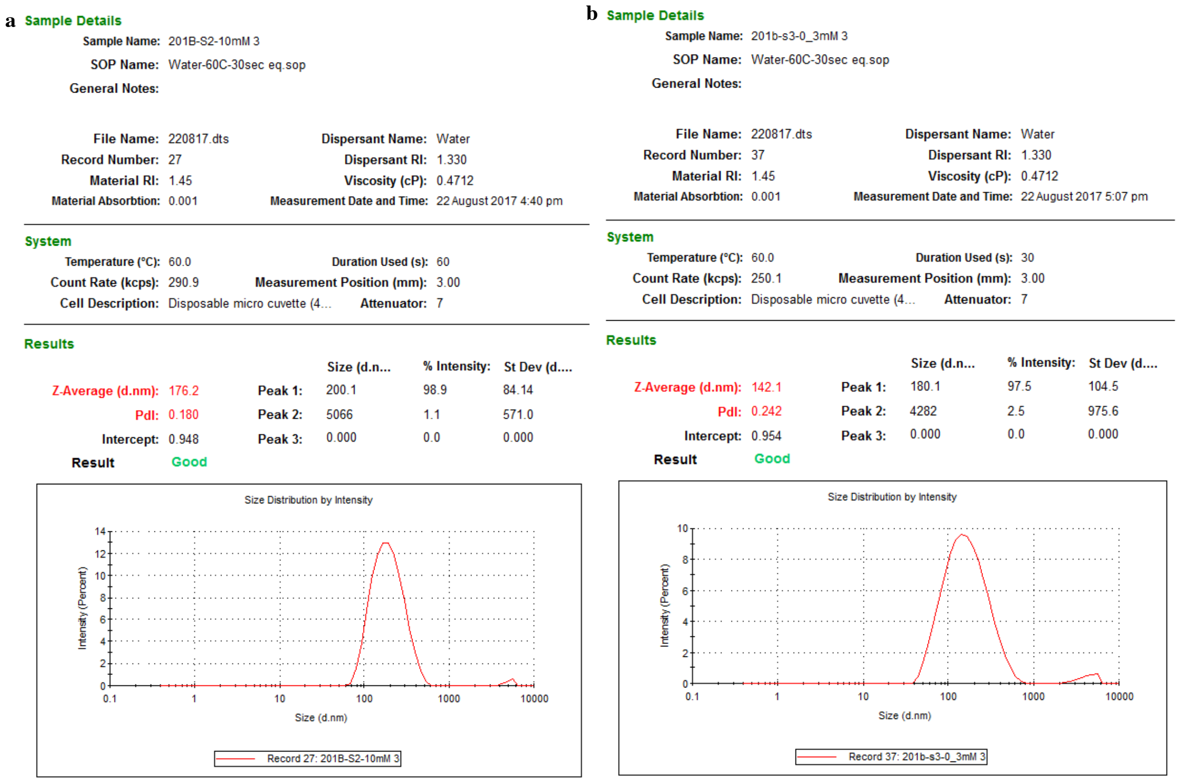

Supplementary Figure 19. DLS experiments using alkene 3b. (a) At 10mM and **(b)** At 0.3mM.

TEM experiments

TEM images were produced using negative staining. 10 μl of sample was applied to freshly glow discharged carbon Formvar 200 mesh copper grids for 2 mins, blotted with filter paper and stained with 2% uranyl acetate for 10 s, then blotted and air dried. Grids were imaged in a FEI Tecnai 12 TEM at 120 kV using a Gatan OneView CMOS camera.

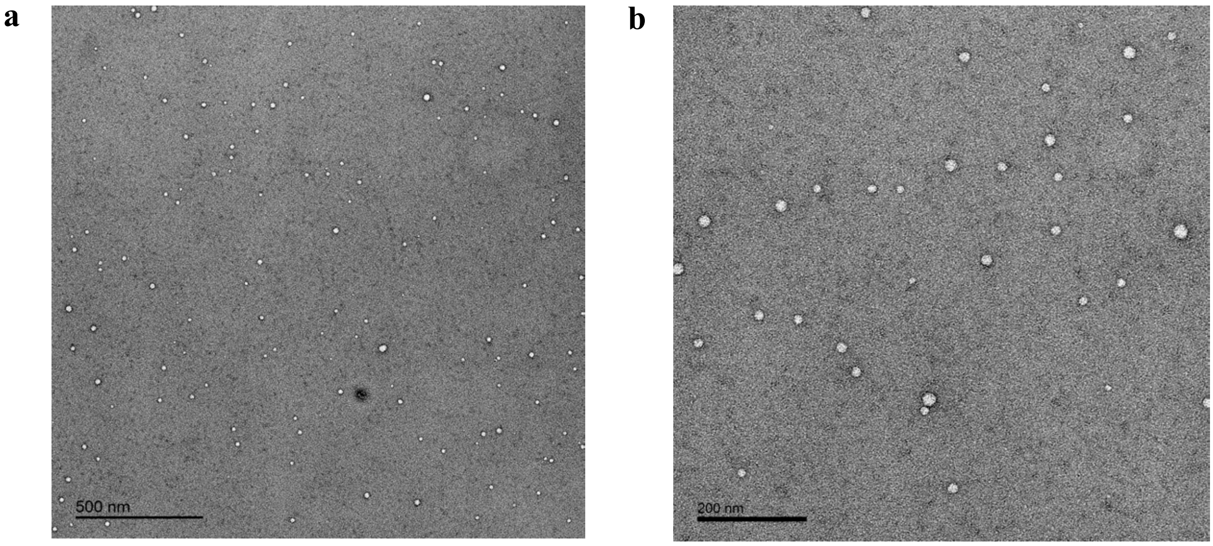

Supplementary Figure 20. TEM images showing particles with a diameter average size of 27nm for amphiphile 3a in a 0.6mM solution. (a) Including a scale bar of 500nm. **(b)** Including a scale bar of 200nm.

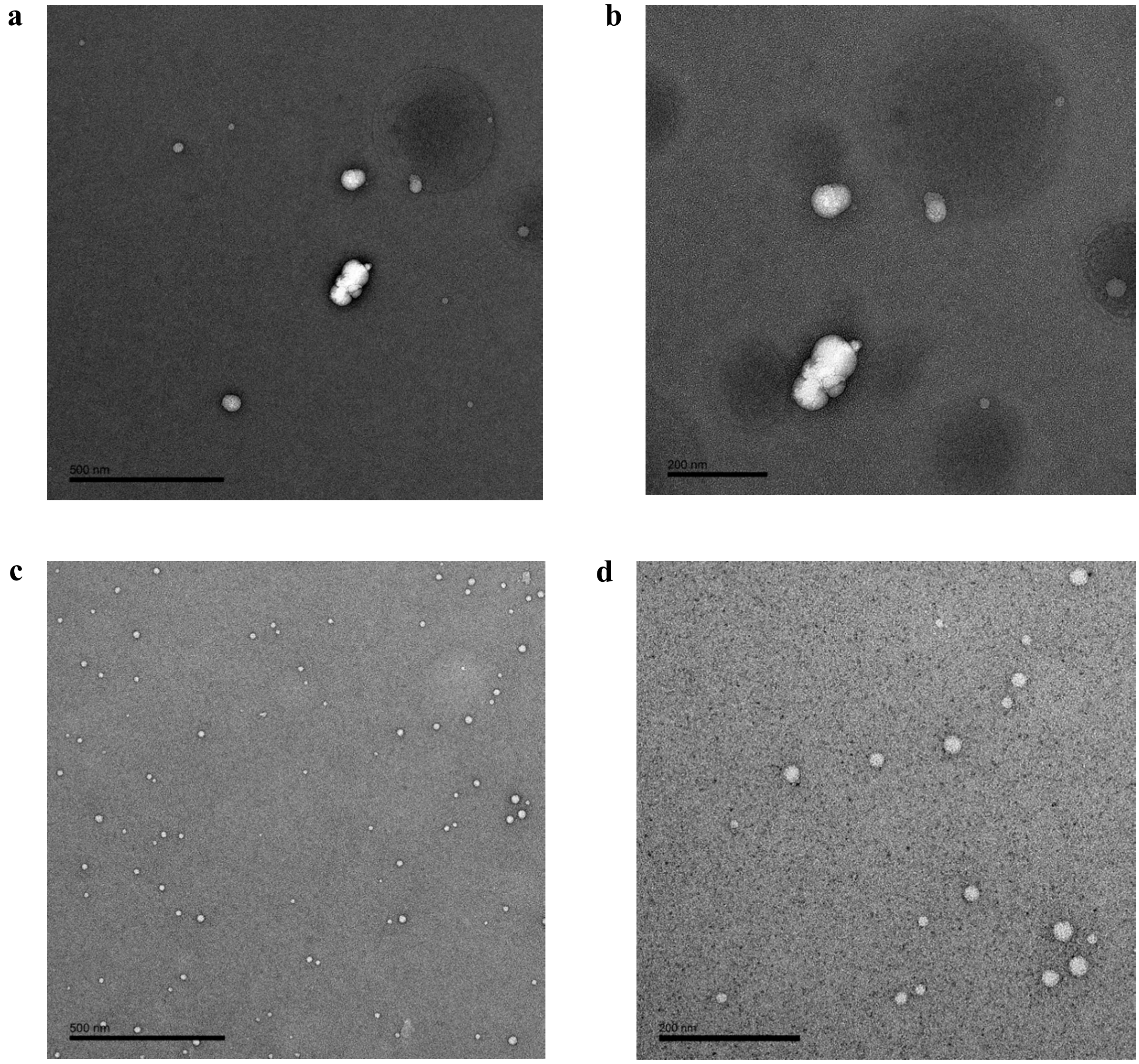

Supplementary Figure 21. TEM images for amphiphile 3b. (a) At a concentration of 2.5mM, aggregates of a 140nm size are observed with a scale bar of 500nm **(b)** or scale bar of 200nm. **(c)** At lower concentration, 0.3mM, smaller particles with a diameter average size of 27nm are detected with a scale bar of 500nm **(d)** or scale bar of 200nm.

NMR spectra

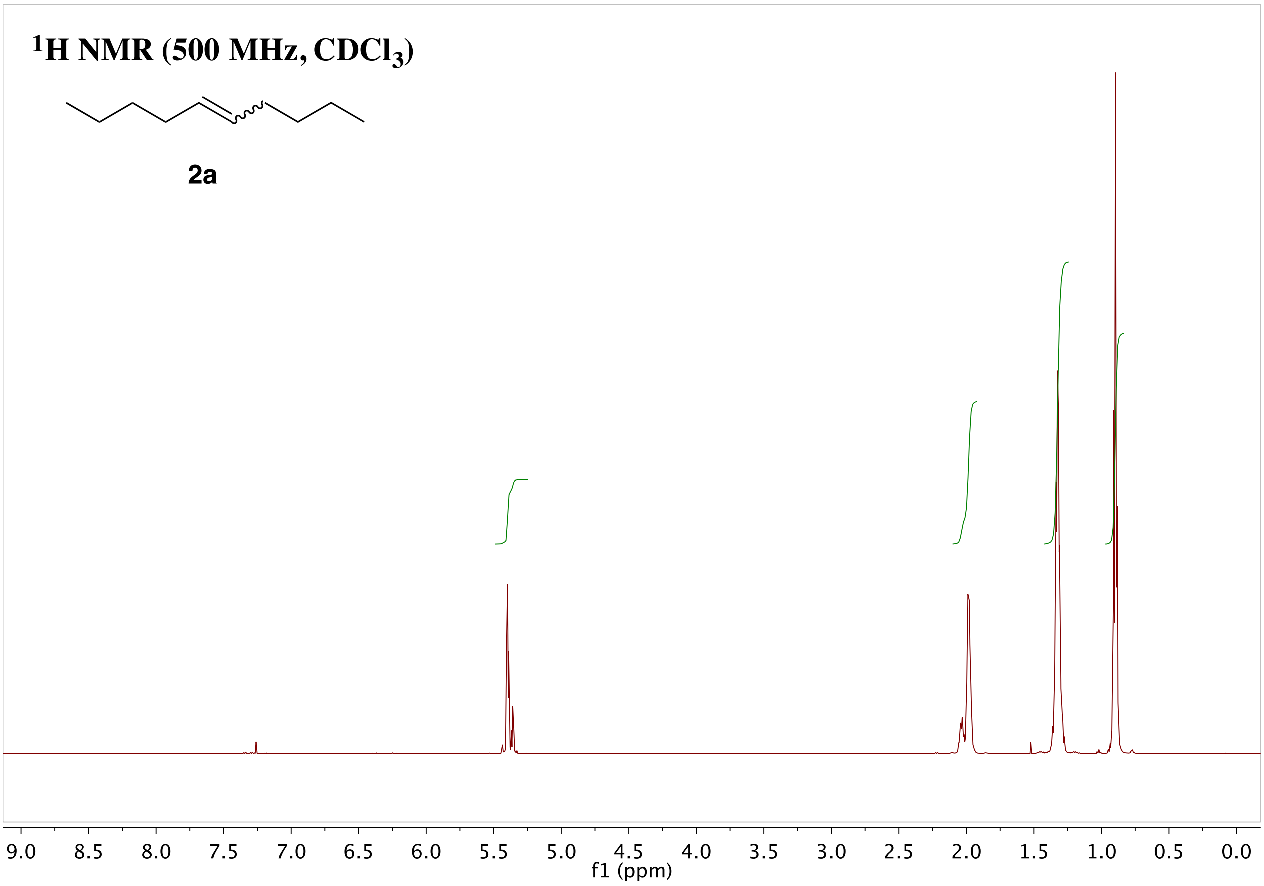

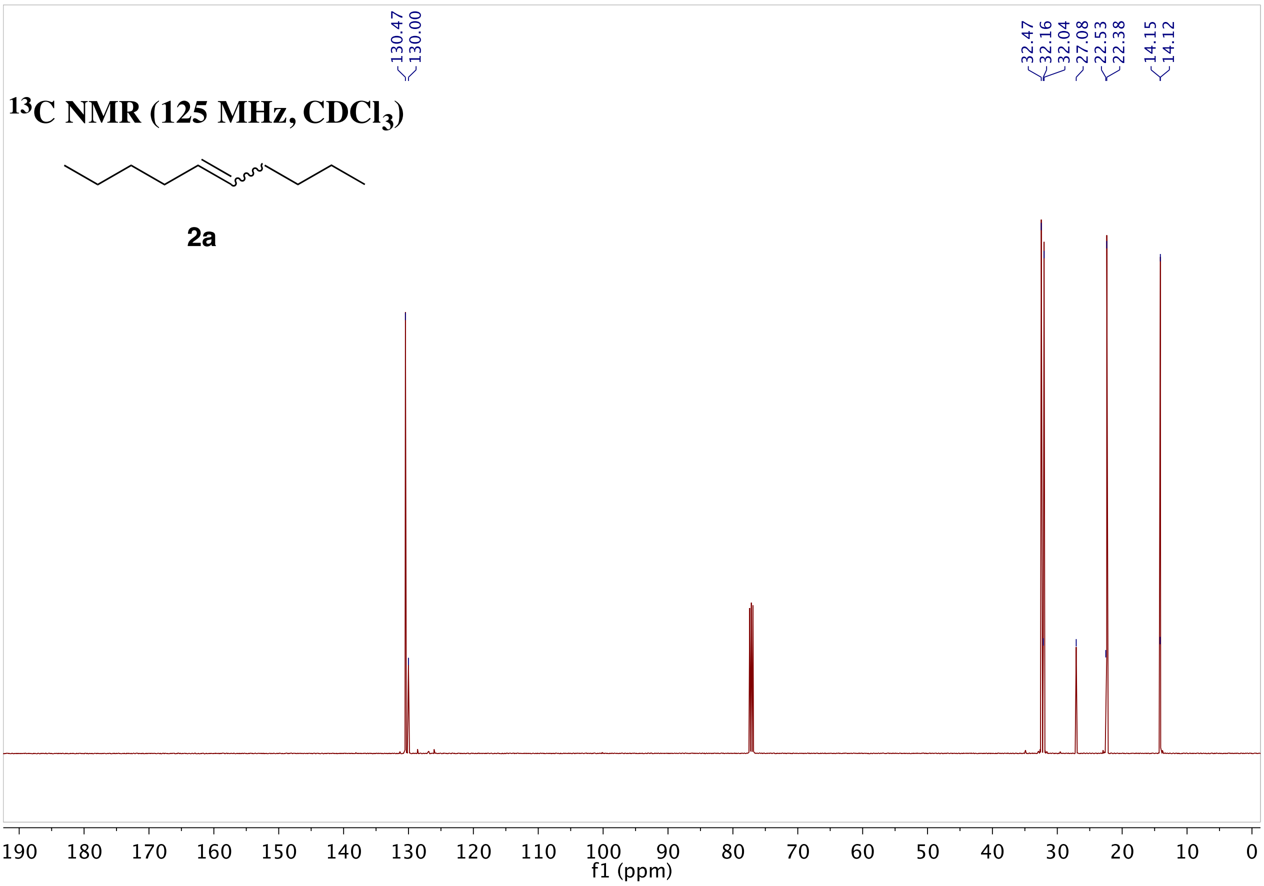

Supplementary Figure 22. ^{1}H NMR and ^{13}C NMR spectra of **2a**.

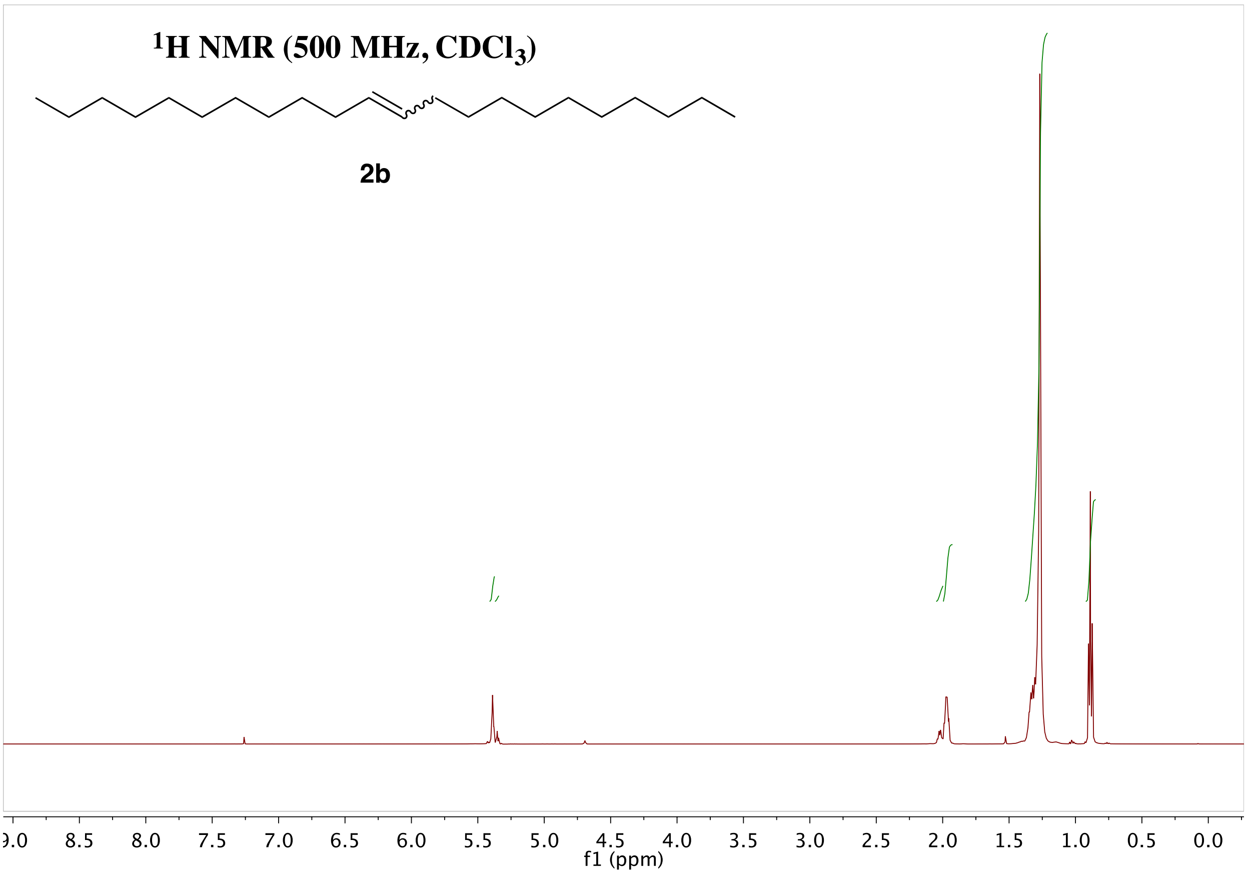

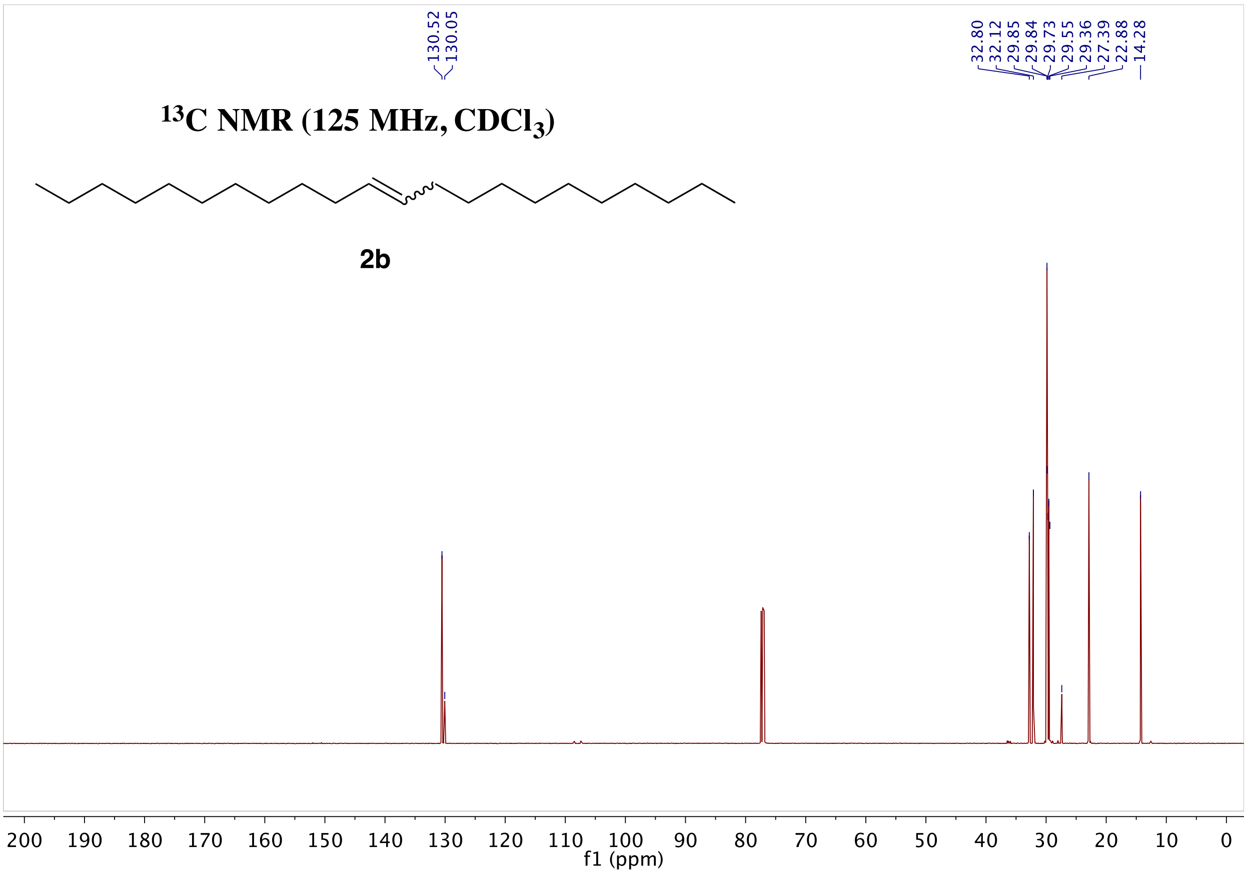

Supplementary Figure 23. ^{1}H NMR and ^{13}C NMR spectra of **2b**.

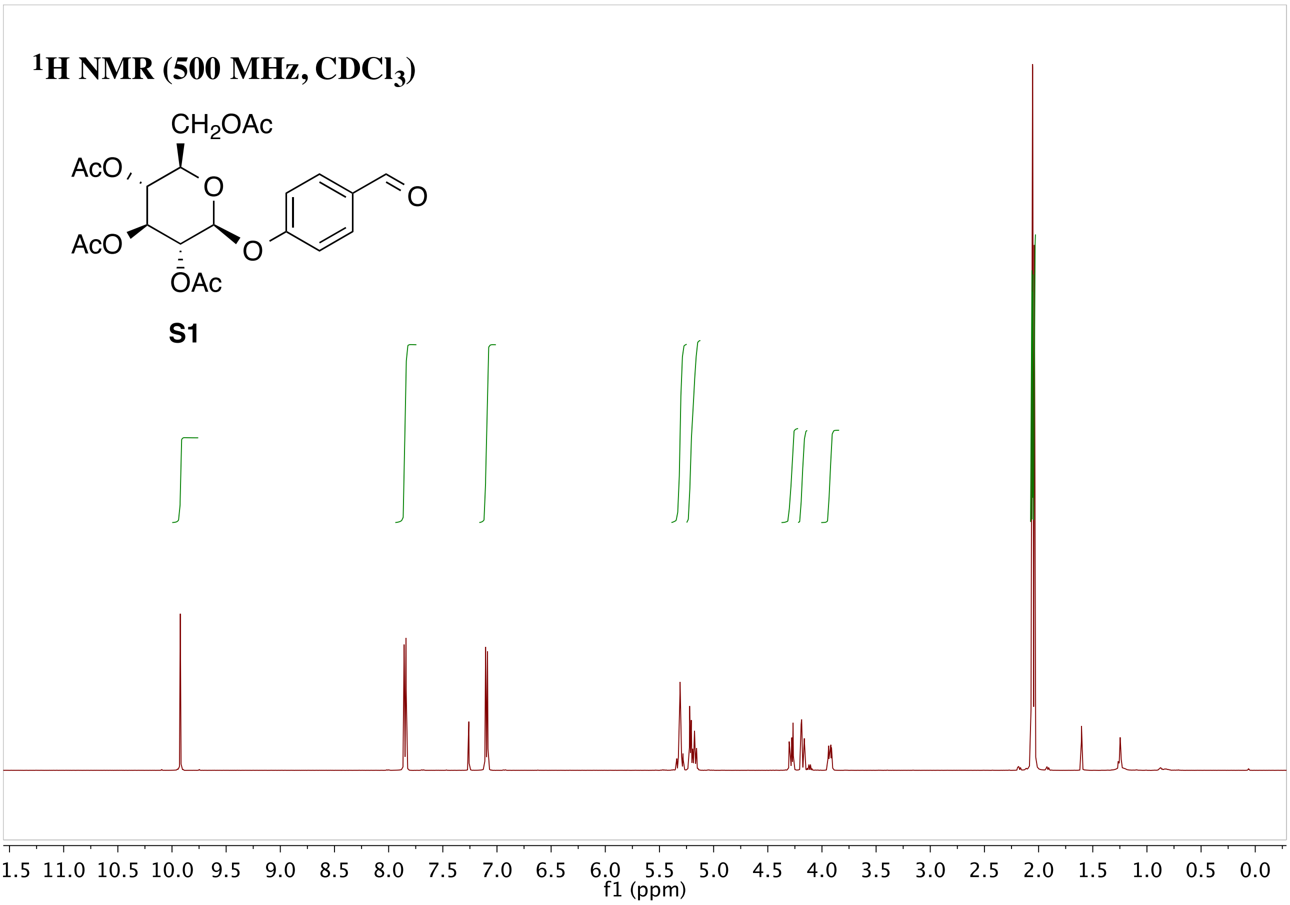

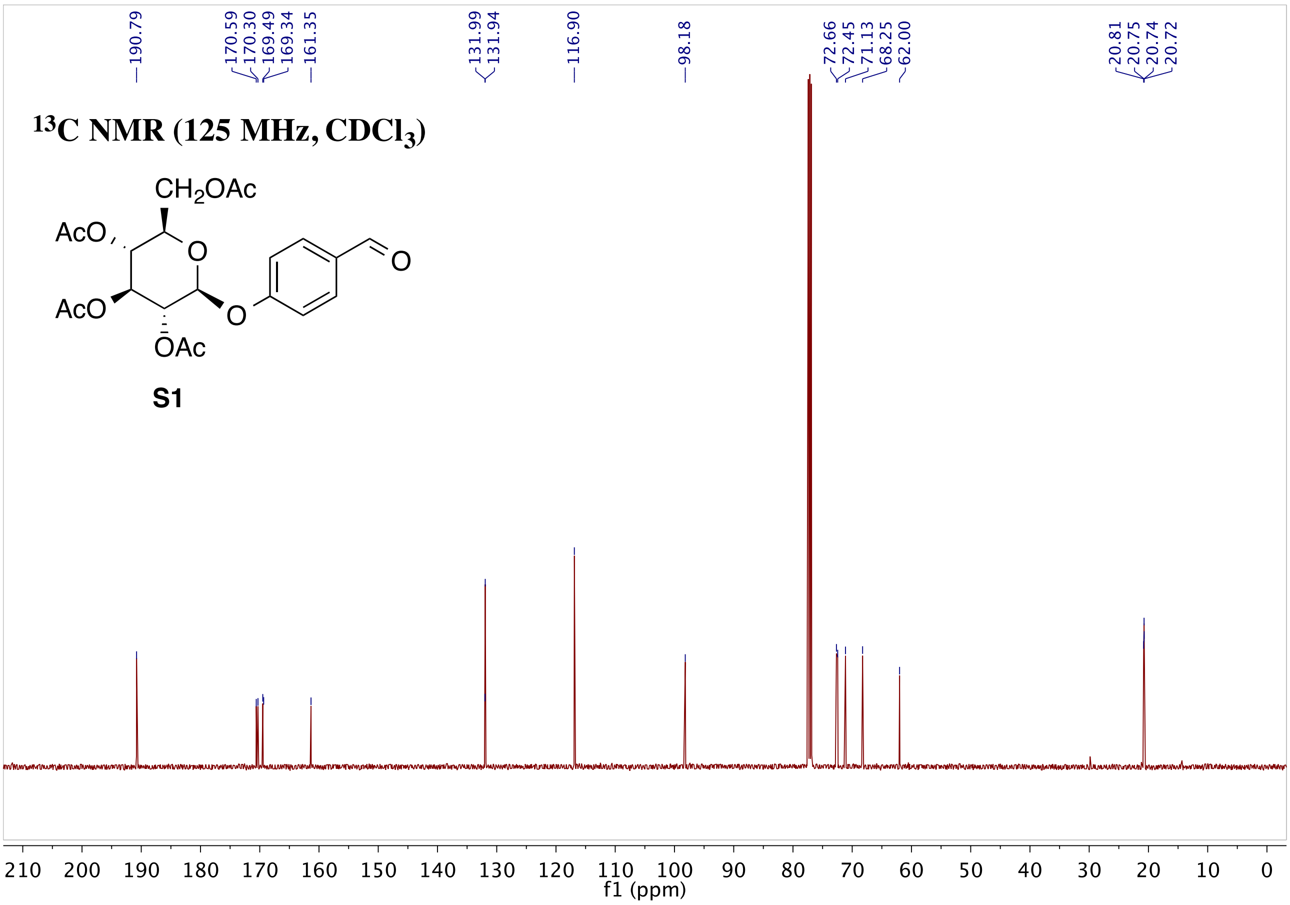

Supplementary Figure 24. ^{1}H NMR and ^{13}C NMR spectra of **S1**.

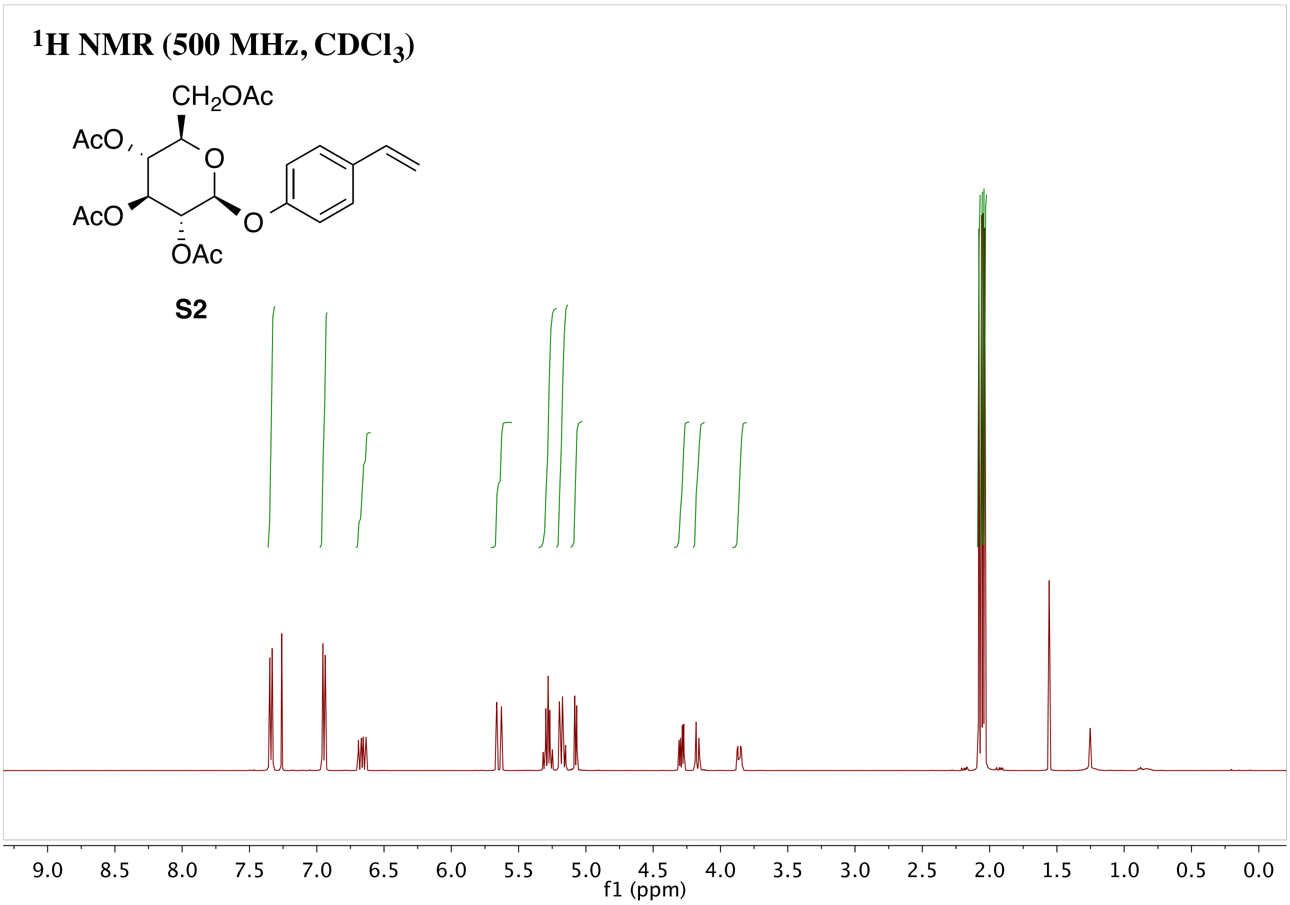

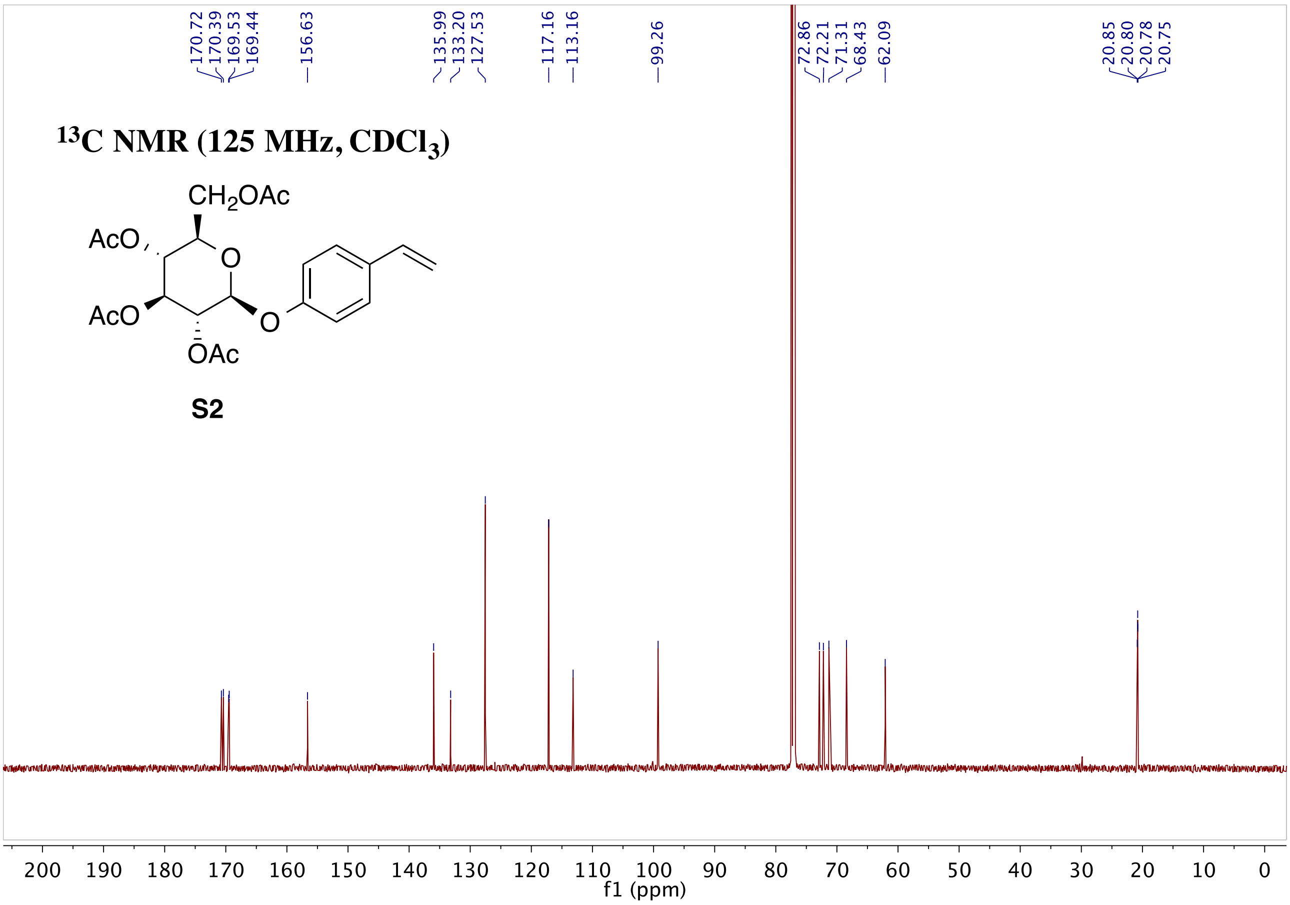

Supplementary Figure 25. ^{1}H NMR and ^{13}C NMR spectra of **S2**.

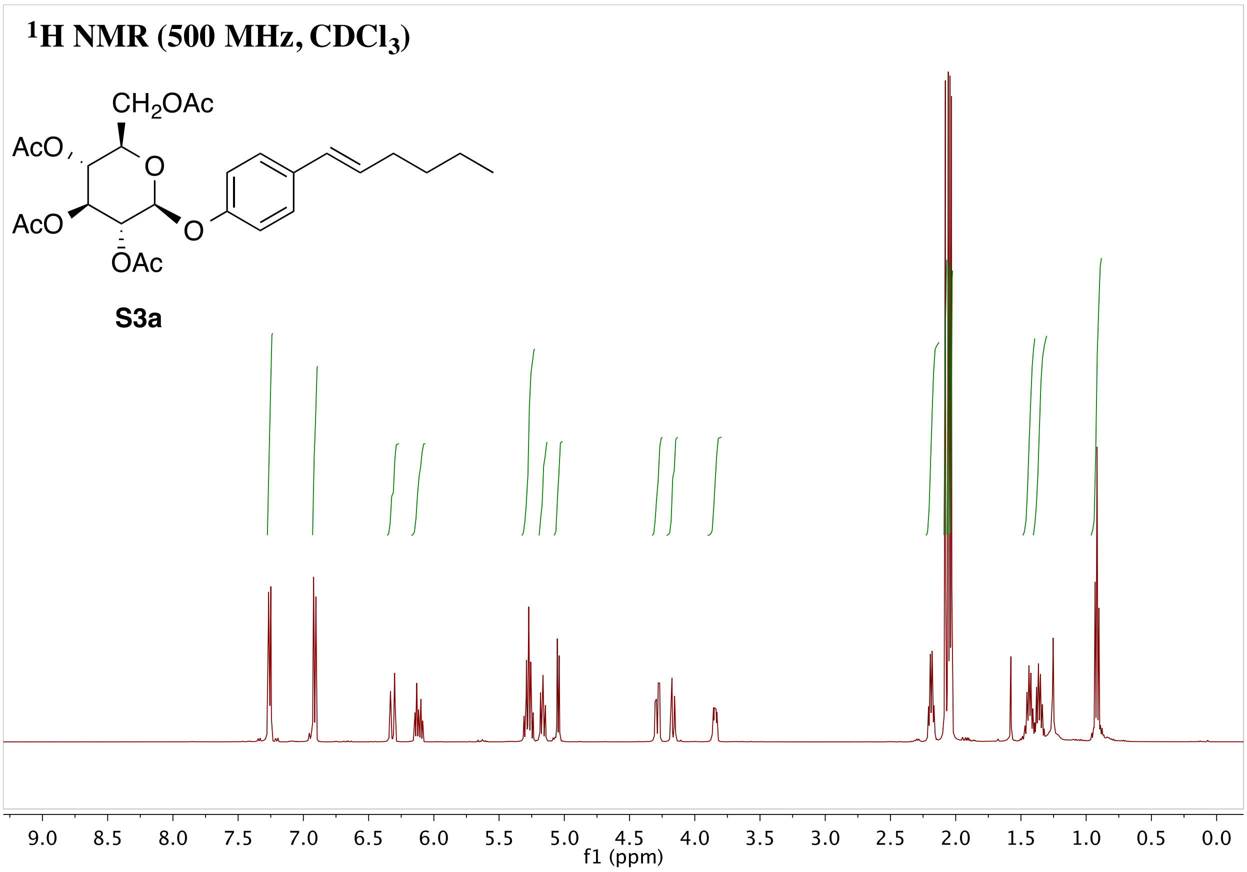

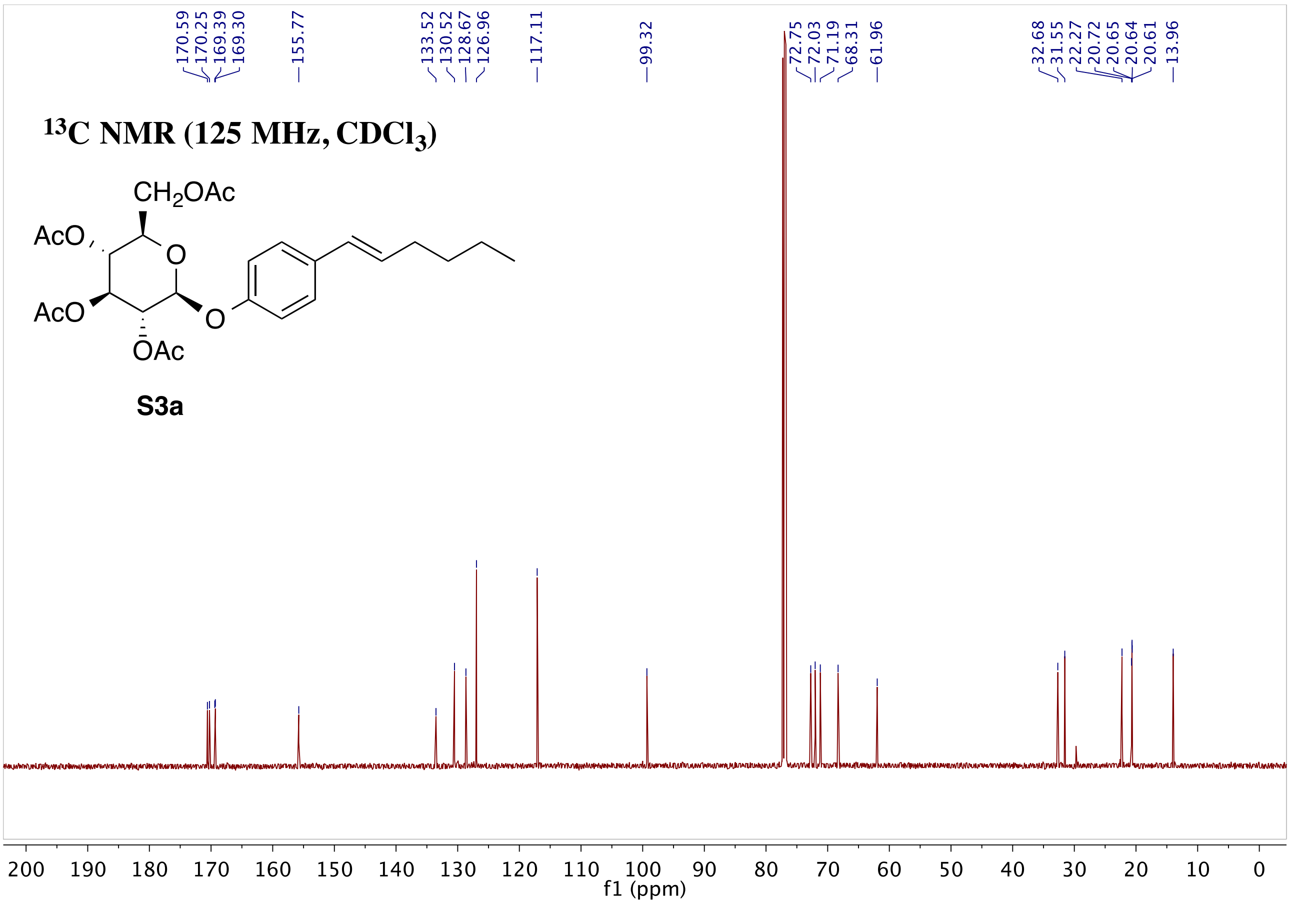

Supplementary Figure 26. ^{1}H NMR and ^{13}C NMR spectra of **S3a**.

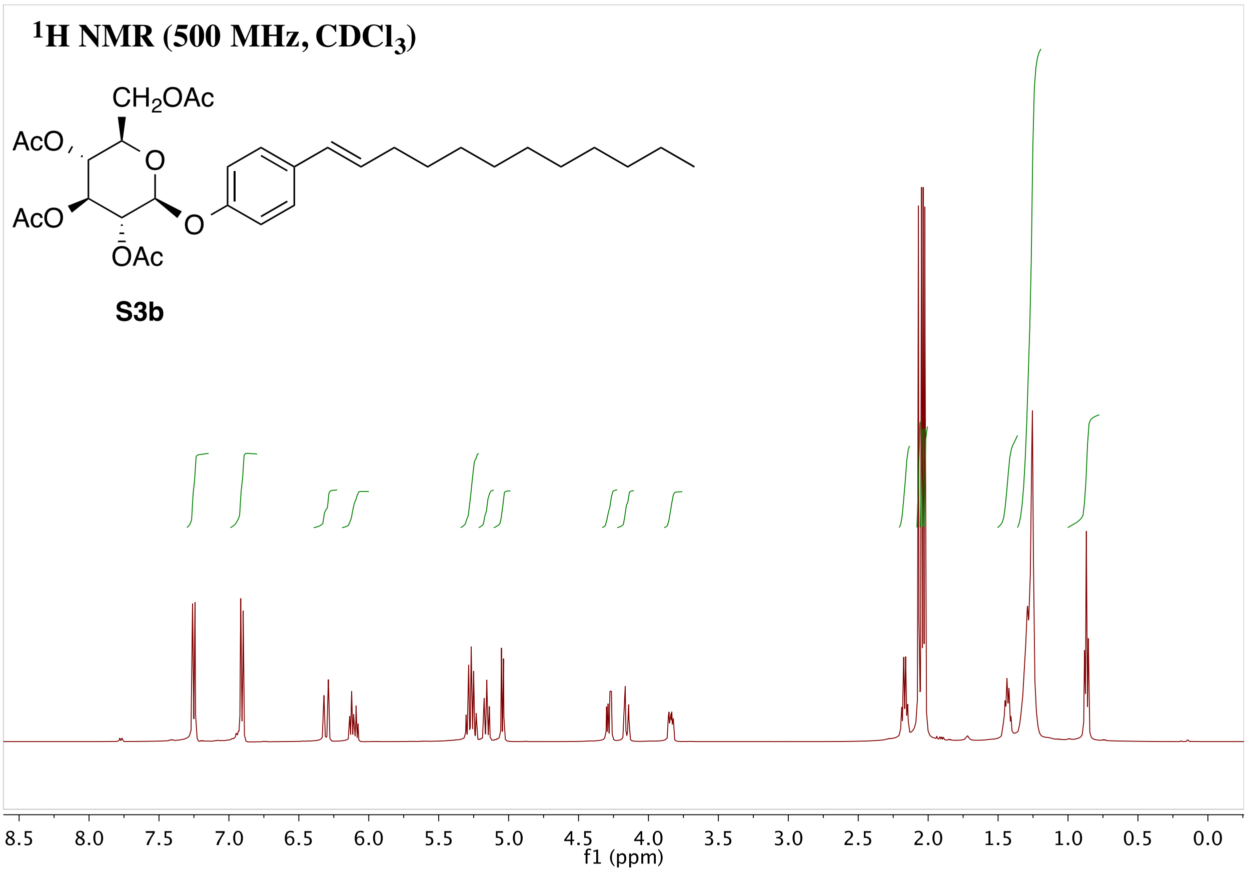

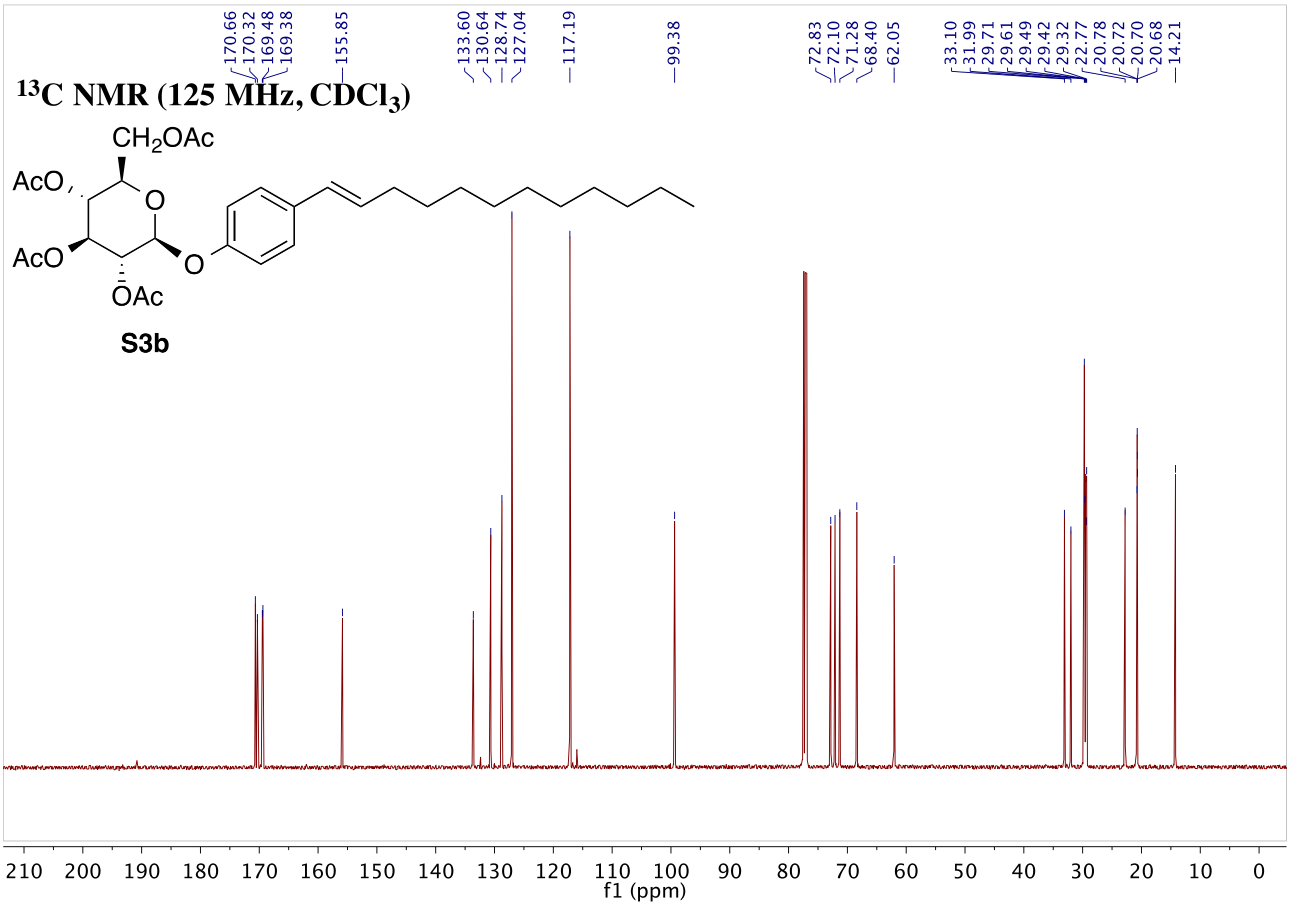

Supplementary Figure 27. ^{1}H NMR and ^{13}C NMR spectra of **S3b**.

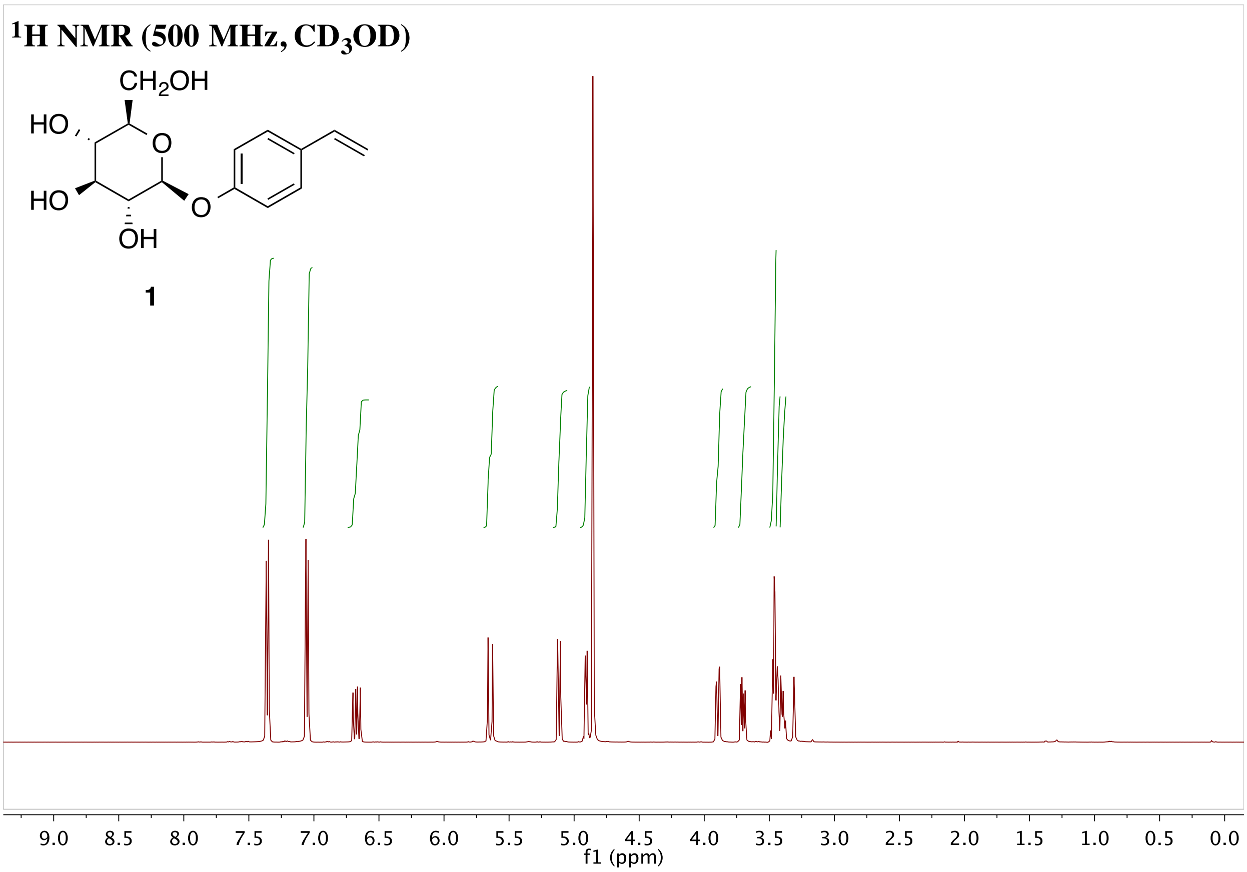

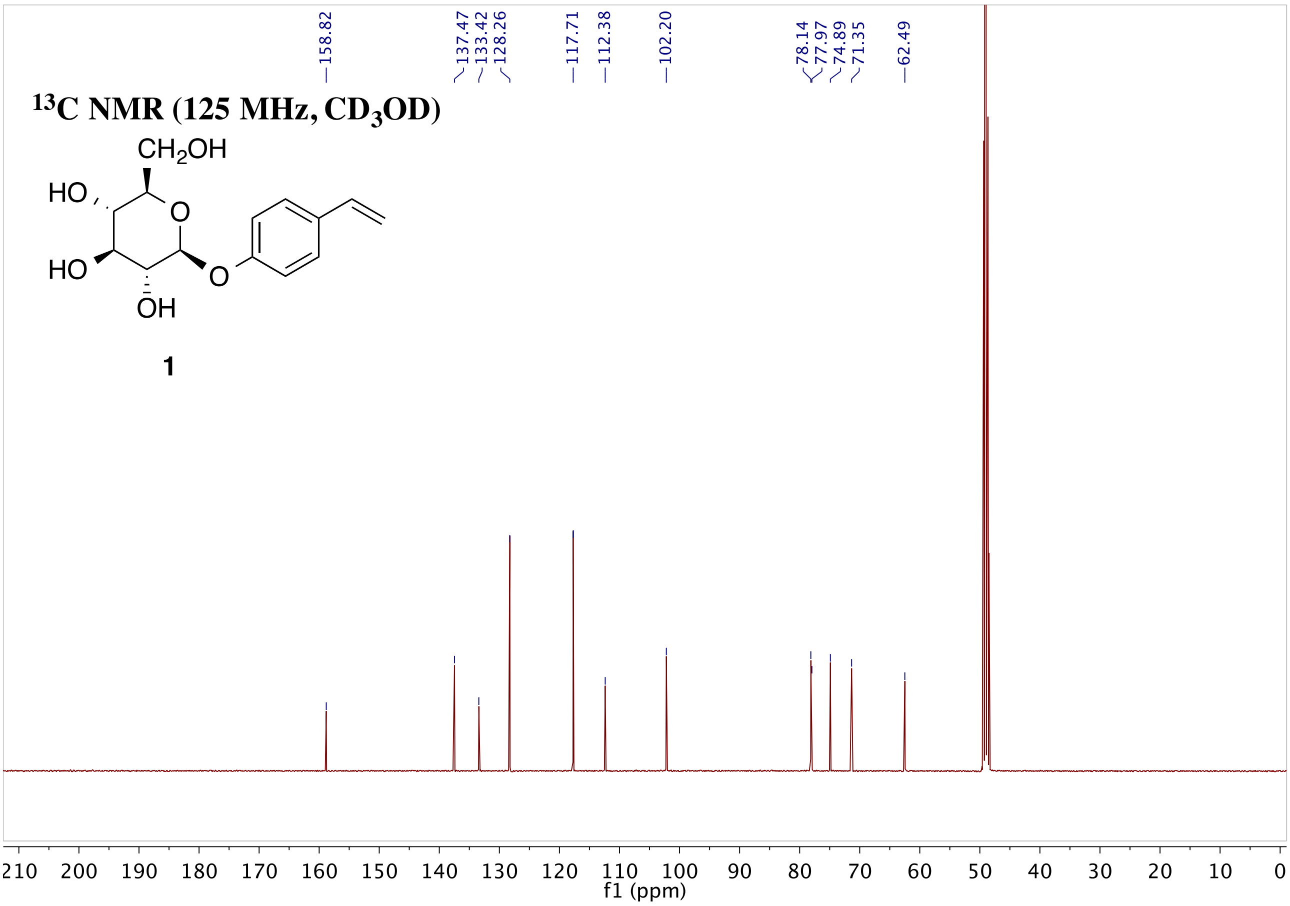

Supplementary Figure 28. ^{1}H NMR and ^{13}C NMR spectra of **1**.

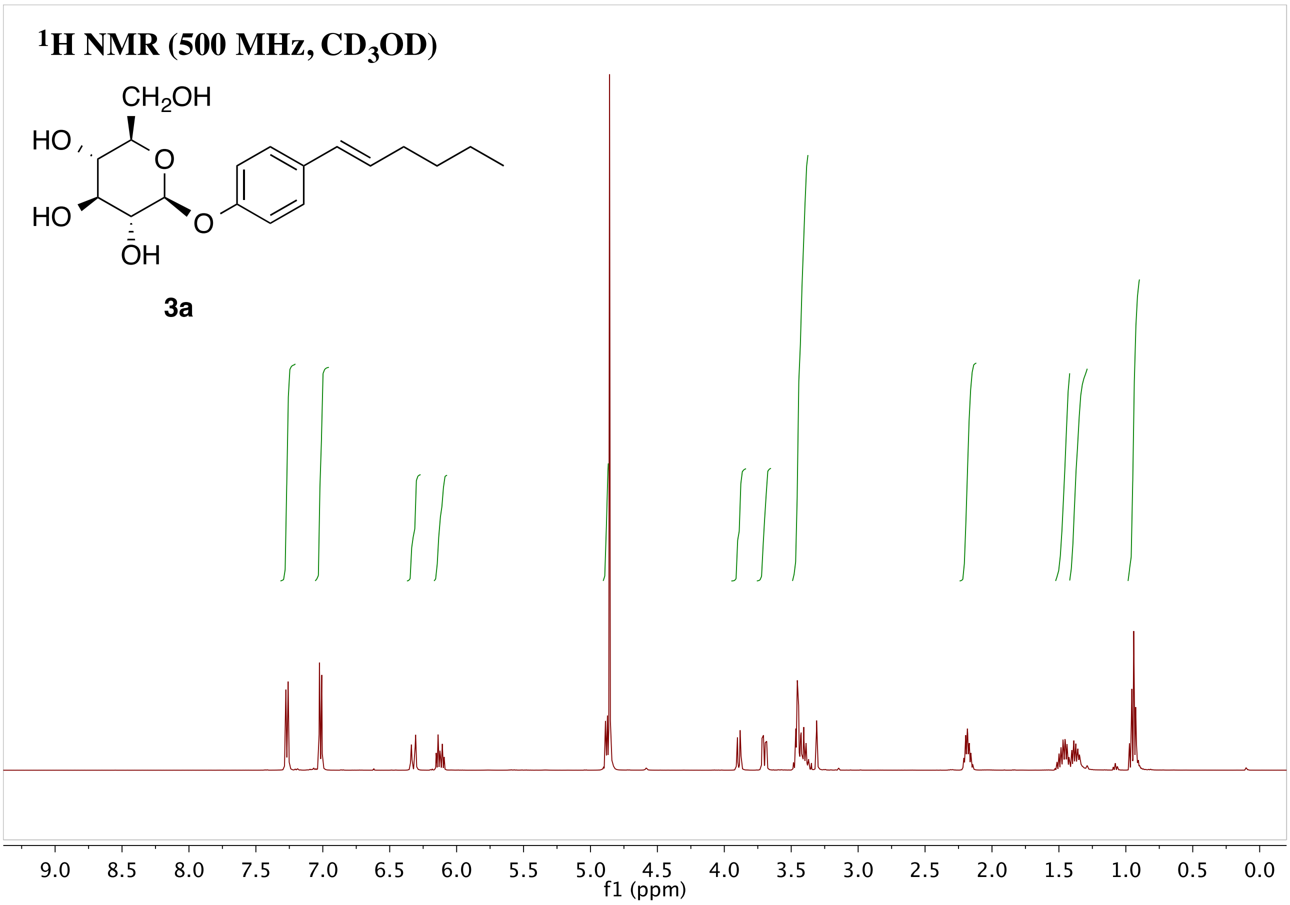

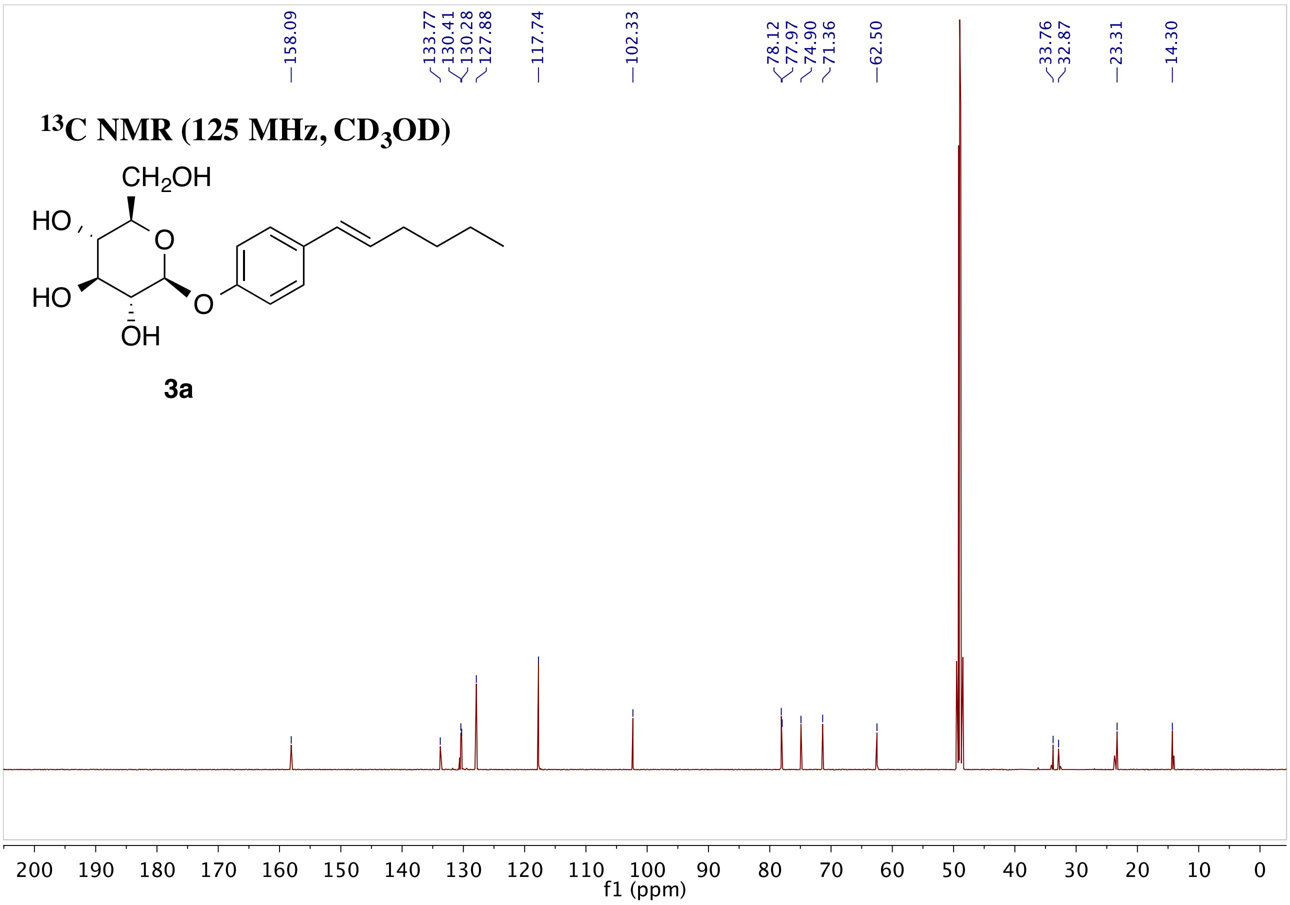

Supplementary Figure 29. ^{1}H NMR and ^{13}C NMR spectra of **3a**.

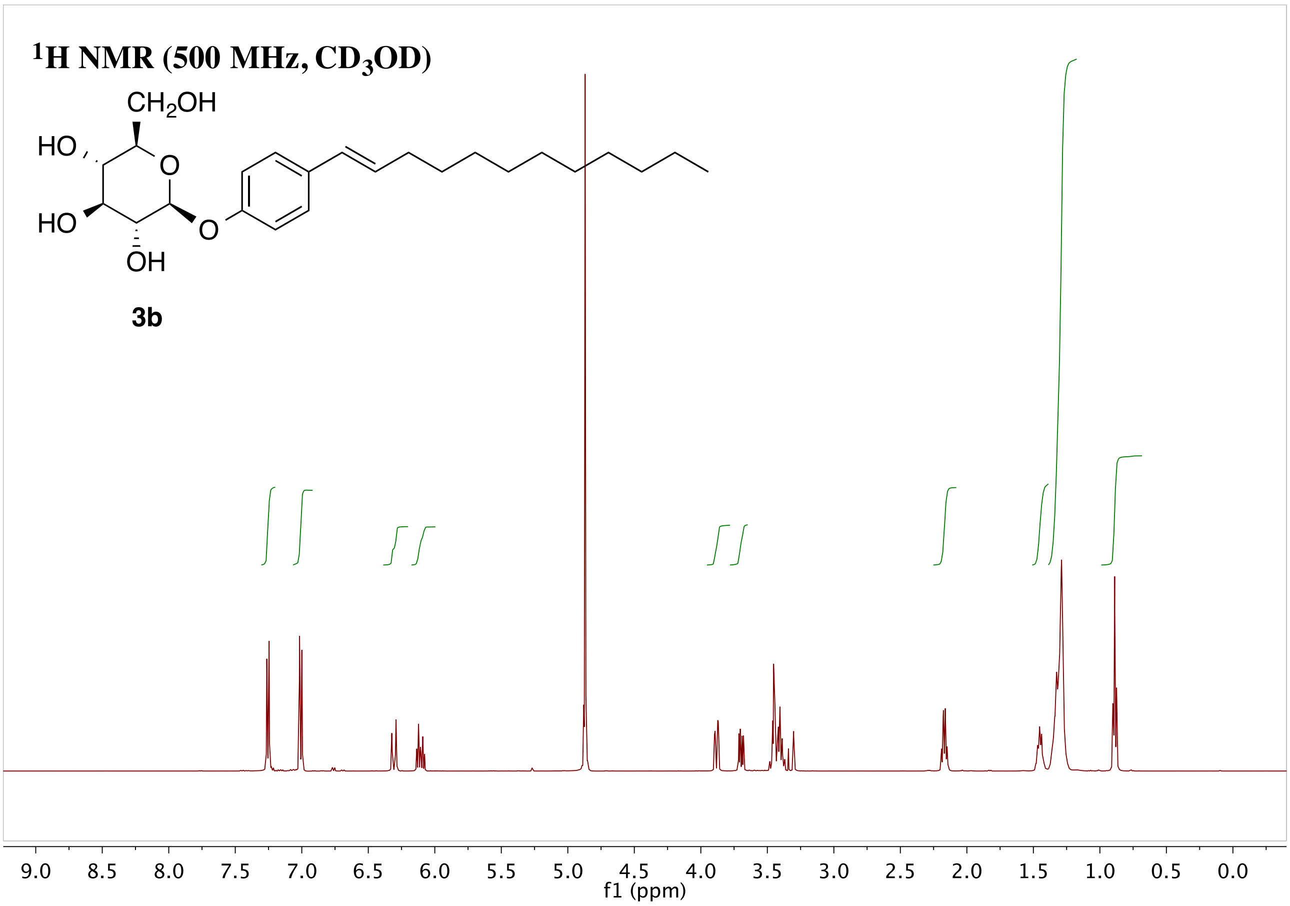

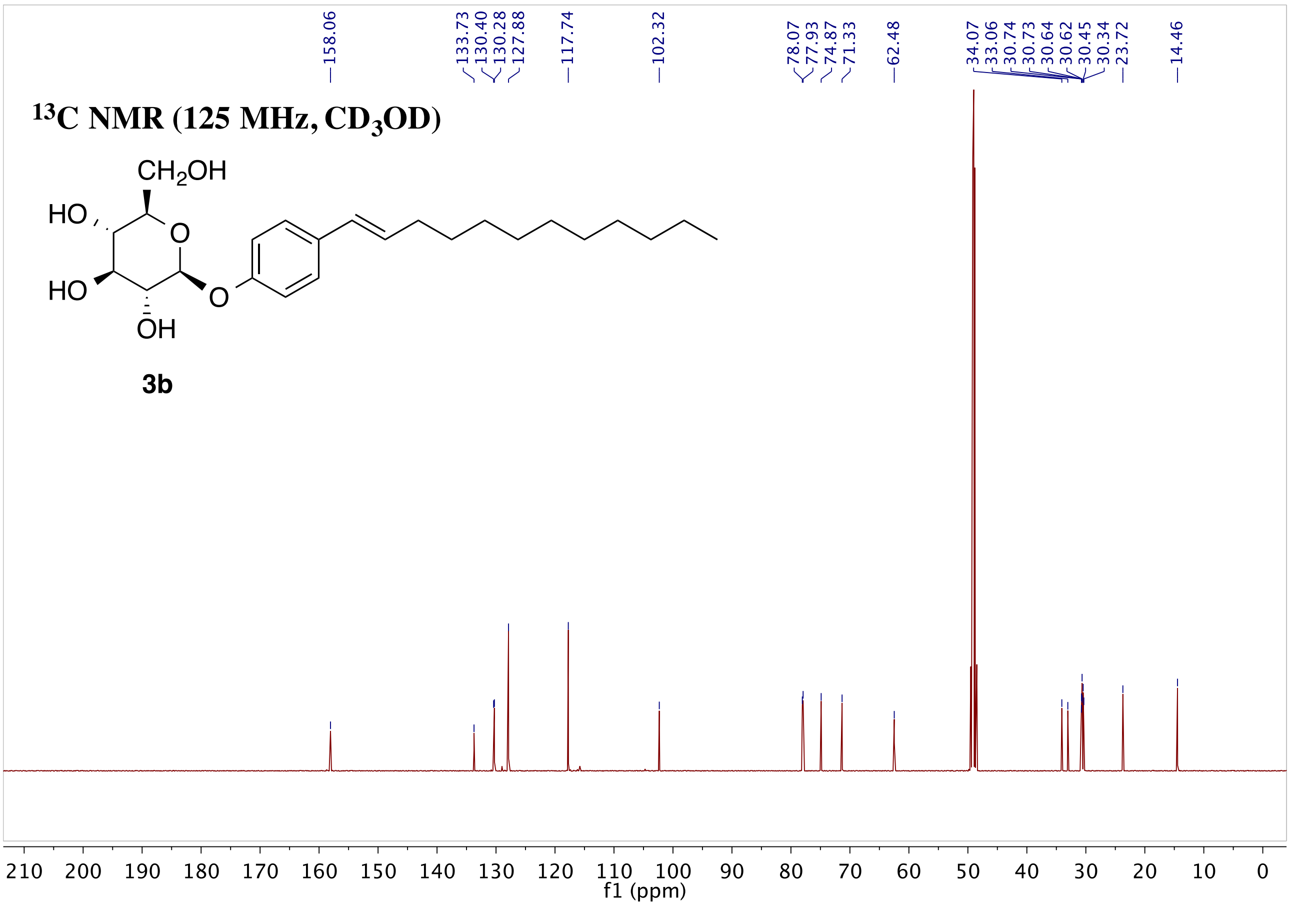

Supplementary Figure 30. ^{1}H NMR and ^{13}C NMR spectra of **3b**.

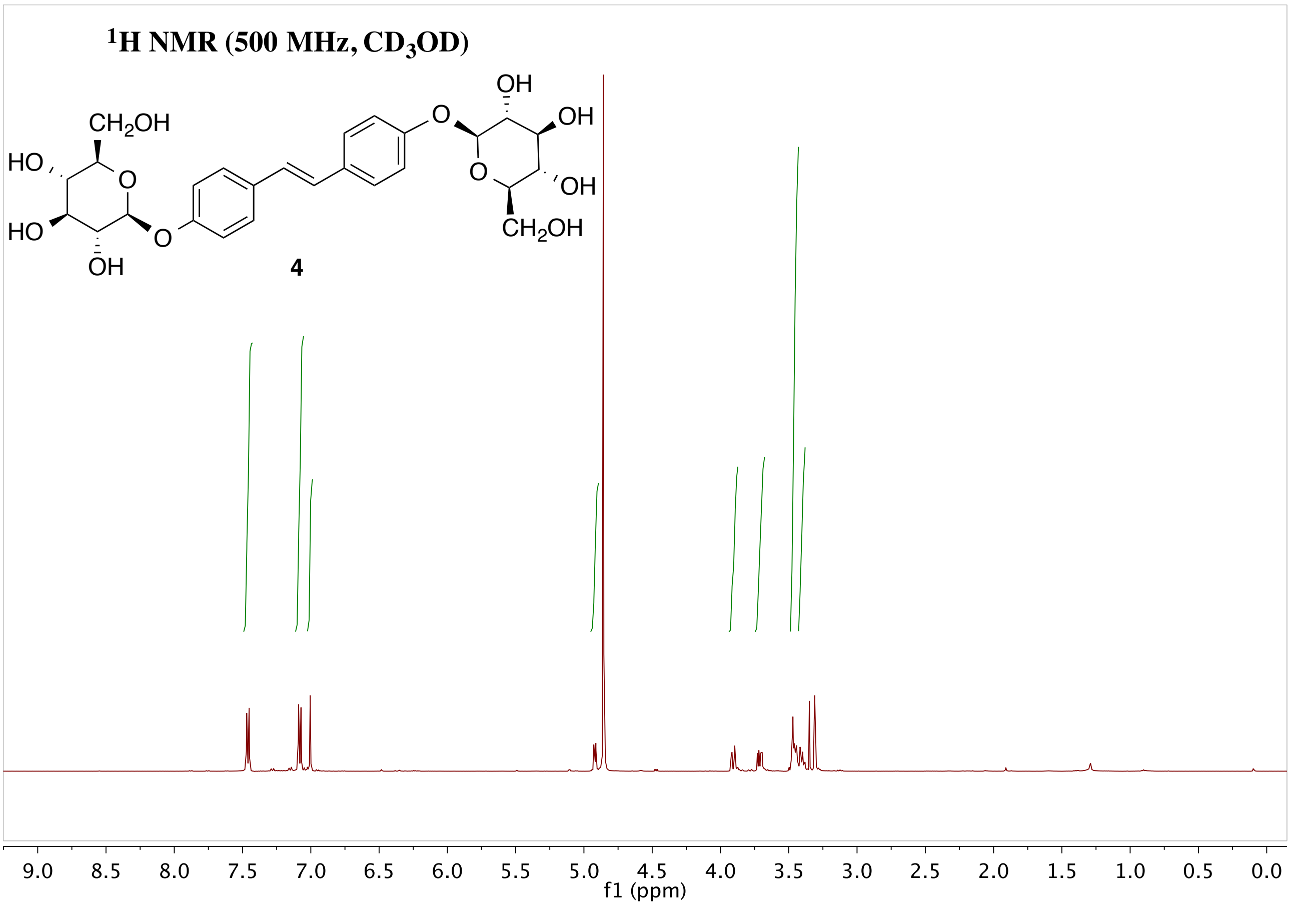

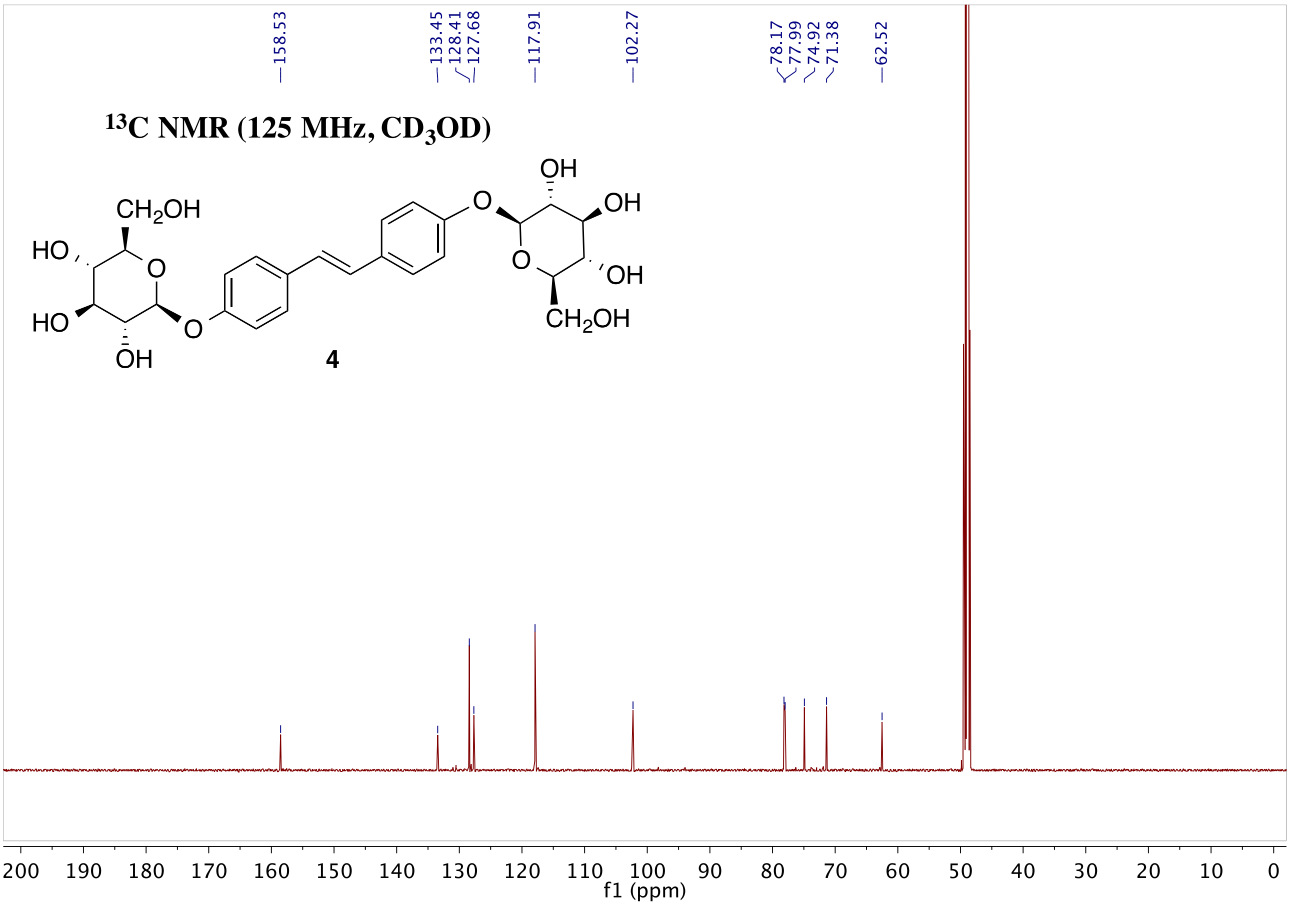

Supplementary Figure 31. ^{1}H NMR and ^{13}C NMR spectra of **4**.

Supplementary references

[1] Jiang, A. J., Zhao, Y., Schrock, R. R. & Hoveyda, A. H. Highly Z-Selective Metathesis Homocoupling of Terminal Olefins. *J. Am. Chem. Soc.* **131**, 16630-16631 (2009).
[2] Czaban, J., Schertzer, B. M. & Grela, K. Low Catalyst Loadings in Self-Metathesis of 1-Dodecene. *Adv. Synth. Catal.* **355**, 1997-2006 (2013).
[3] Oulmi, D., Maillard, P., Guerquin-Kern, J.-L., Huel, C. & Momenteau, M. Glycoconjugated Porphyrins. 3. Synthesis of Flat Amphiphilic Mixed meso-(Glycosylated aryl)arylporphyrins and Mixed meso-(Glycosylated aryl)alkylporphyrins Bearing Some Mono- and Disaccharide Groups. *J. Org. Chem.* **60**, 1554-1564 (1995).
[4] Chattopadhyay, A. & London, E. Fluorimetric determination of critical micelle concentration avoiding interference from detergent charge. *Anal Biochem* **139**, 408-412 (1984).
